# Tertiary siRNAs Mediate Paramutation in *C*. *elegans*

**DOI:** 10.1371/journal.pgen.1005078

**Published:** 2015-03-26

**Authors:** Alexandra Sapetschnig, Peter Sarkies, Nicolas J. Lehrbach, Eric A. Miska

**Affiliations:** 1 Wellcome Trust Cancer Research UK Gurdon Institute, University of Cambridge, Cambridge, United Kingdom; 2 Department of Biochemistry and Department of Genetics, University of Cambridge, Cambridge, United Kingdom; Brandeis University, United States of America

## Abstract

In the nematode *Caenorhabditis elegans*, different small RNA-dependent gene silencing mechanisms act in the germline to initiate transgenerational gene silencing. Piwi-interacting RNAs (piRNAs) can initiate transposon and gene silencing by acting upstream of endogenous short interfering RNAs (siRNAs), which engage a nuclear RNA interference (RNAi) pathway to trigger transcriptional gene silencing. Once gene silencing has been established, it can be stably maintained over multiple generations without the requirement of the initial trigger and is also referred to as RNAe or paramutation. This heritable silencing depends on the integrity of the nuclear RNAi pathway. However, the exact mechanism by which silencing is maintained across generations is not understood. Here we demonstrate that silencing of piRNA targets involves the production of two distinct classes of small RNAs with different genetic requirements. The first class, secondary siRNAs, are localized close to the direct target site for piRNAs. Nuclear import of the secondary siRNAs by the Argonaute HRDE-1 leads to the production of a distinct class of small RNAs that map throughout the transcript, which we term tertiary siRNAs. Both classes of small RNAs are necessary for full repression of the target gene and can be maintained independently of the initial piRNA trigger. Consistently, we observed a form of paramutation associated with tertiary siRNAs. Once paramutated, a tertiary siRNA generating allele confers dominant silencing in the progeny regardless of its own transmission, suggesting germline-transmitted siRNAs are sufficient for multigenerational silencing. This work uncovers a multi-step siRNA amplification pathway that promotes germline integrity via epigenetic silencing of endogenous and invading genetic elements. In addition, the same pathway can be engaged in environmentally induced heritable gene silencing and could therefore promote the inheritance of acquired traits.

## Author Summary

Transgenerational epigenetic gene silencing has been shown to be important for organisms to react directly to their environment without the need to acquire genetic mutations. The inheritance of acquired traits via the gametes can prove advantageous in fast reproducing organisms. In *Caenorhabditis elegans*, a free-living nematode, multigenerational epigenetic inheritance can be induced by exogenous (experimentally provided) and endogenous cues that trigger small RNA-dependent gene silencing in the germline of these animals. PIWI interacting small RNAs (piRNAs) are required for the initiation of stable silencing of invading genomic elements in the germline such as transposons. Gene silencing established by piRNAs can subsequently be maintained over multiple generations without the original trigger. In *C*. *elegans*, this stable maintenance of silencing requires an additional class of small interfering RNAs (siRNAs) that must be amplified in each generation in order to maintain multigenerational silencing. Here we show that these siRNAs fall into two distinct classes, which we call secondary and tertiary siRNAs. We find that the production of tertiary siRNAs is part of a nuclear amplification pathway associated with the stable heritable silencing of an allele, a form of paramutation. This amplification pathway therefore promotes germline integrity and possibly the inheritance of acquired physiological traits.

## Introduction

Transgenerational epigenetic inheritance is the transmission of information from parent to offspring via the gametes by means other than the primary sequence of DNA [1]. A number of recent examples have highlighted the possibility for epigenetic inheritance of acquired traits in animals. This resurrection of Larmarckism is poised to rewrite the textbook on heredity [2], but a clearer understanding of the molecular mechanisms involved is needed.

In the nematode *Caenorhabditis elegans*, epigenetic inheritance of acquired traits can be triggered by exposure to environmental RNA. In these cases, gene silencing mediated by the RNA interference (RNAi) pathway is maintained for multiple generations independently of the initial trigger [3–5]. RNAi is thought to have evolved as a system of surveillance for harmful nucleic acids, and acts to eliminate parasitic nucleic acids such as those derived from viral infection. As such, this epigenetic inheritance pathway may provide in inherited ‘immunity’ against viral infection, or other harmful environmentally derived RNAs.

Small RNA pathways related to RNAi also play an important role in defending the genome from endogenous parasitic nucleic acids, such as transposons, retrotransposons, and endogenous retroviruses. Silencing of these elements in germline cells is essential to ensure genome stability, and faithful transmission of genetic material to the next generation [6]. Piwi interacting small RNAs (piRNAs) are a class of germline-specific small RNAs that are required for transposon silencing, genome stability and fertility in animals, and have been linked to epigenetic inheritance in a number of species, including the mouse, *Drosophila* and *C*. *elegans* [7]. In this case, epigenetic inheritance may analogously provide an inherited immunity to the potentially harmful nucleic acids harboured within animal genomes.

Piwi proteins are a subclass of the Argonaute family of proteins (Ago), a central component of small RNA pathways. Ago proteins physically associate with small, 21–35 nucleotide-long RNAs thereby forming a complex whose specificity is determined by base pairing interactions between the small RNA and target nucleic acids. This typically leads to repression of gene expression, which can occur by various mechanisms [8]. The *C*. *elegans* Piwi homologue (PRG-1), associates with piRNAs (also termed 21U-RNAs in *C*. *elegans* for their characteristic length and 5′ nucleotide) and directs silencing of transposons and some endogenous genes (“piRNA targets”) in the germline, and is important for fertility [9,10]. PRG-1 triggers silencing of piRNA targets via a small RNA amplification pathway [9]. Target recognition occurs in the cytoplasm by base-pairing between a piRNA and target mRNA and prompts the generation of secondary siRNAs (termed 22G-RNAs for their characteristic length and 5′ nucleotide) antisense to the target mRNA. This process involves the RNA dependent RNA polymerases (RDRPs) RRF-1 and EGO-1, the Dicer-related helicase DRH-3 and a number of proteins in the Mutator (Mut) class (including MUT-2,-7,-16) [11], and is thought to take place in perinuclear structures in the germline termed Mutator foci [12]. Efficient silencing of piRNA targets by 22G-RNAs requires the nuclear RNAi pathway, consisiting of the nuclear RNAi factors NRDE-1, -2 and -4 and the germline-specific nuclear Argonaute HRDE-1, which binds to 22G-RNAs [3,13]. Nuclear RNAi is thought to inhibit gene expression by interfering with transcription, possibly at the elongation stage [14] and may do so by promoting a chromatin state associated with gene silencing [15]. Consistent with a role for repressive histone modifications, efficient silencing of piRNA targets requires the HP1 orthologue HPL-2, and the putative histone methyltransferases SET-32 and SET-25 [3].

Importantly, although PRG-1 is required to initiate biogenesis of 22G-RNAs antisense to piRNA targets, both 22G-RNAs and target gene silencing can be stably maintained over multiple generations independently of PRG-1 [3]. Similarly, 22G-RNAs and silencing initiated by ingested dsRNA can be maintained in the germline for multiple generations without ingestion of dsRNA [3,13,16,17]. Transgenerational maintenance of silencing requires nuclear RNAi and chromatin factors. However, as these components are required as effectors of silencing by RNAi, it is unclear which of these factors play an active role in transmission of epigenetic information between generations.

An important observation in defining the inherited signal behind epigenetic inheritance of RNAi-mediated silencing is that transgenerational silencing is associated with maintenance of high levels of 22G-RNAs, even several generations after exposure to the initial trigger [3,4]. Thus, understanding the mechanism of transgenerational RNAi requires elucidation of how 22G-RNAs are amplified across generations. Considering the large number of progeny generated each generation, this must reflect synthesis of new siRNAs. However, such amplification would suggest that secondary 22G-RNAs can drive synthesis of additional similar (tertiary) small RNAs. As yet there is no direct evidence for tertiary siRNAs biogenesis in the germline of *C*. *elegans*. Indeed, a recent study characterizing siRNAs generated in response to exogenous dsRNA suggested that secondary 22G-RNAs have limited ability to initiate a tertiary siRNA response under the conditions tested [18]. Thus there is a conflict between the observations of stable 22G-RNA populations and the lack of evidence for a mechanism that could generate them. Understanding this is crucial to understanding how small RNAs are involved in the maintenance of epigenetic states.

In this study, we gain important insight into the resolution of this paradox by addressing the mechanism whereby 22G-RNAs are generated at piRNA target genes. We find that, in fact, a tertiary 22G-RNA population is found at piRNA target genes. Tertiary 22G-RNAs are formed downstream of secondary 22G-RNAs and are not directly dependent on initial target recognition. Crucially, this population is dependent on the activity of the nuclear RNAi pathway, in particular requiring the engagement of the secondary 22G-RNAs with the nuclear Argonaute HRDE-1 for their formation. Additionally, we find that these 22G-RNAs are necessary and sufficient to instigate a form of paramutation [19] whereby a stably silenced allele is generated that can in turn silence further alleles in *trans* independently of the initial trigger. Our work reveals a feed-forward siRNA amplification loop that guides epigenetic inheritance in animals.

## Results

### 22G-RNAs distal to piRNA target sites require the nuclear RNAi pathway

**Fig 1:**
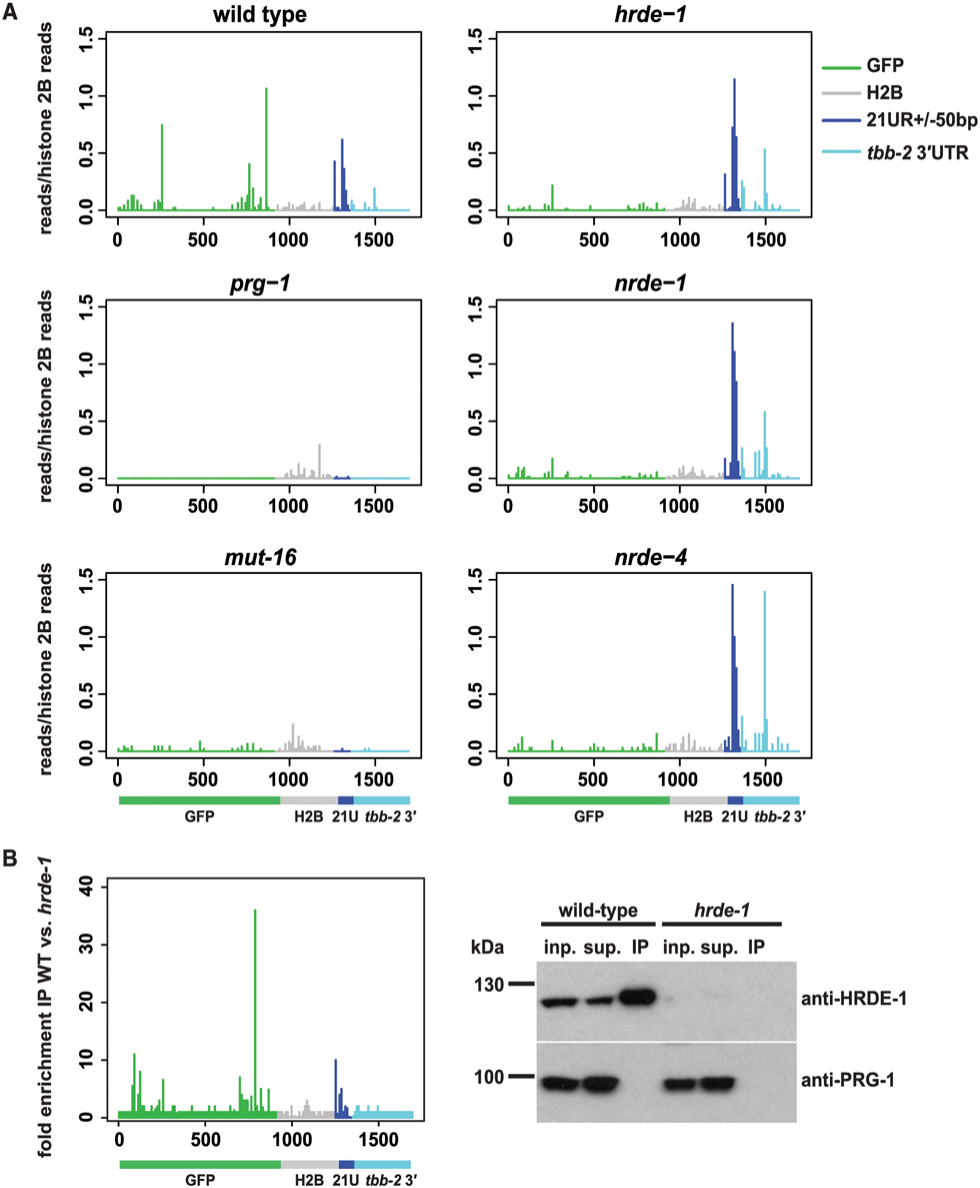
22G-RNAs distal to piRNA target sites require the nuclear RNAi pathway. A) Small RNA high-throughput sequencing reads with unique matches antisense to the *piRNA sensor* from wild type and various mutant animals as indicated. The values of the *y*-axes correspond to reads matching the *piRNA sensor* normalised to reads matching Histone 2B (*his-58*). The x-axes represent the relative position of reads in the *piRNA sensor* transgene with numbers representing nucleotides from the start codon (set as 0). The transgene structure is schematically represented at the bottom. Colour code: green = GFP, grey = Histone 2B (*his-58*), dark blue = 21UR-1 target site plus/minus 50 bp, light blue = *tbb-2* 3′UTR. B) Left panel: Enrichment of small RNA high-throughput sequencing reads with matches antisense to the *piRNA sensor* in HRDE-1 Immunoprecipitation (IP). Displayed is the fold enrichment of reads found in anti-HRDE-1 IP from wild type versus *hrde-1* mutant animals. X-axis, schematic representation of the transgene and colour code as in A). Right panel: Western blot of anti-HRDE-1 Immunoprecipitation from wild type (left 3 lanes) and *hrde-1* mutant (right 3 lanes) animals. Antibodies used for western blot are anti-HRDE-1 and anti-PRG-1 as loading control. Inp. = 1% input, sup. = supernatant, IP = Immuoprecipitate. On the left, the relative migration of the 130 and 100 kDa bands of the PAGE Ruler Plus (MBI Fermentas) marker are indicated.

We and others previously showed that the nuclear RNAi pathway is required for silencing of piRNA targets in *C*. *elegans* [3,4]. Utilizing a reporter transgene consisting of a GFP sequence upstream of a target site for the endogenous *C*. *elegans* piRNA 21UR-1 (the *piRNA sensor*), we were able to detect secondary siRNAs generated near the piRNA target site in *nrde-1* mutant animals [3], suggesting nuclear RNAi acts downstream of secondary siRNA synthesis to silence piRNA targets. To investigate the role of the nuclear RNAi pathway more carefully, we carried out deep sequencing of small RNAs in wildtype and nuclear RNAi defective strains carrying the *piRNA sensor*. To our surprise, we found that although proximal 22G-RNAs localizing to within 200bp of the piRNA target site are still present, distal 22Gs mapping to the remainder of the GFP coding sequence (as observed in wild type animals, Fig. 1A) are lost in *hrde-1*, *nrde-1* and *nrde-4* mutants. In contrast, *prg-1* mutants lack both proximal and distal 22G-RNAs against the *piRNA sensor* mRNA, as do mutants lacking the mutator protein MUT-16, which is required for synthesis of many 22G-RNAs (Fig. 1A).

The requirement for the nuclear RNAi machinery suggested that 22G-RNAs mapping to the *piRNA sensor* might bind to the nuclear Argonaute HRDE-1. To test this we sequenced small RNAs associated with HRDE-1 using immunoprecipitation with a specific antibody (Fig. 1B). Both proximal and distal 22G-RNAs were strongly enriched in the immunoprecipitated HRDE-1 complexes compared to a negative control immunoprecipitation (Fig. 1B). Since only the distal 22G-RNAs were absent in *hrde-1* mutants (Fig. 1A), this implies that HRDE-1 binding to 22G-RNAs mapping close to the piRNA target site is required for formation of 22G-RNAs mapping across the transcript.

The generation of 22G-RNAs requires RdRPs that utilise a targeted mRNA as a template. We therefore performed high-throughput sequencing of small RNAs from animals mutant in RdRPs carrying the *piRNA sensor* or an alternative *mCherry piRNA sensor* transgene and mapped 22G-RNA antisense reads to the transgene. As compared to wild type animals, single gene mutants of *rrf-1*, *rrf-2*, *rrf-3* and *ego-1* showed no reduction in either class of 22G-RNAs (S1 Fig). However, the double mutant of *rrf-1* and *ego-1* showed a severe reduction of both proximal and distal 22G-RNAs mapping to the *piRNA sensor*, consistent with the observation that mutations in both genes are necessary to desilence a piRNA target [11]. In addition to RdRPs, the Dicer-related helicase DRH-3 was reported to be essential for the production of most 22G-RNAs. Initial high-throughput sequencing of a helicase-dead mutant of *drh-3* suggested a function for DRH-3 in the 3′ to 5′ spreading of 22G-RNAs across endogenous genes [20] reminiscent of our observations in the *nrde* mutants. However, when using the same helicase mutant line to analyse small RNAs mapping antisense to the *piRNA sensor*, we observed loss of both proximal and distal 22G-RNAs, analogous to the *rrf-1 ego-1* double mutant (S1 Fig). Thus neither the helicase domain of DRH-3 nor one particular RdRP is required specifically for the generation of the distal 22G-RNAs.

**Fig 2:**
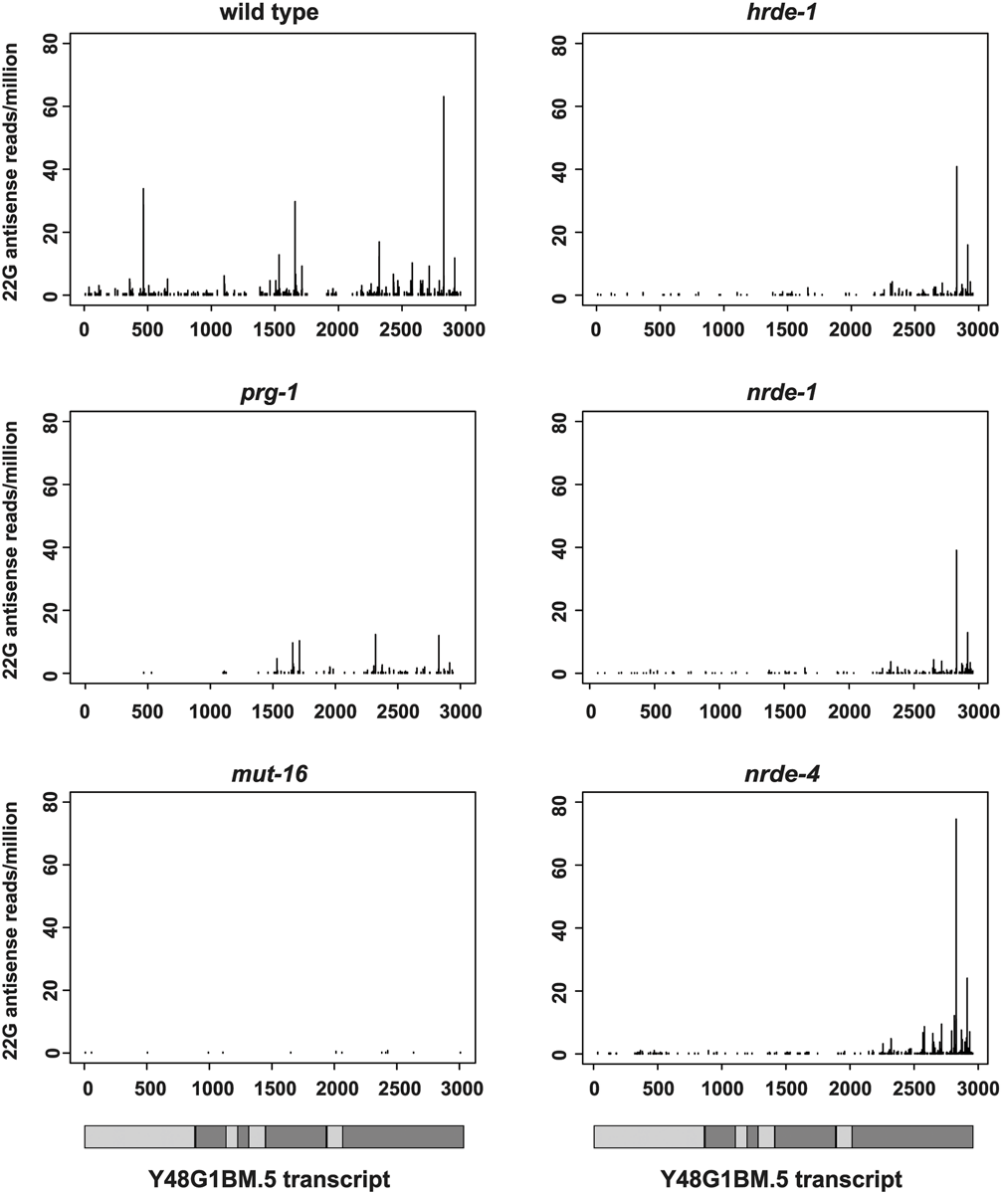
An endogenous target shows 3′ to 5′ spreading of 22G-RNAs. Small RNA high-throughput sequencing reads with unique matches antisense to Y48G1B8M.5 from wild type and mutant animals as indicated. The values of the *y*-axes are antisense 22G siRNA reads per million of total reads. The x-axes represent the relative position of reads in the target gene with numbers representing nucleotides from the start codon (set as 0). The transcript structure is schematically depicted at the bottom with light grey and dark grey boxes representing alternating exons.

**Fig 3:**
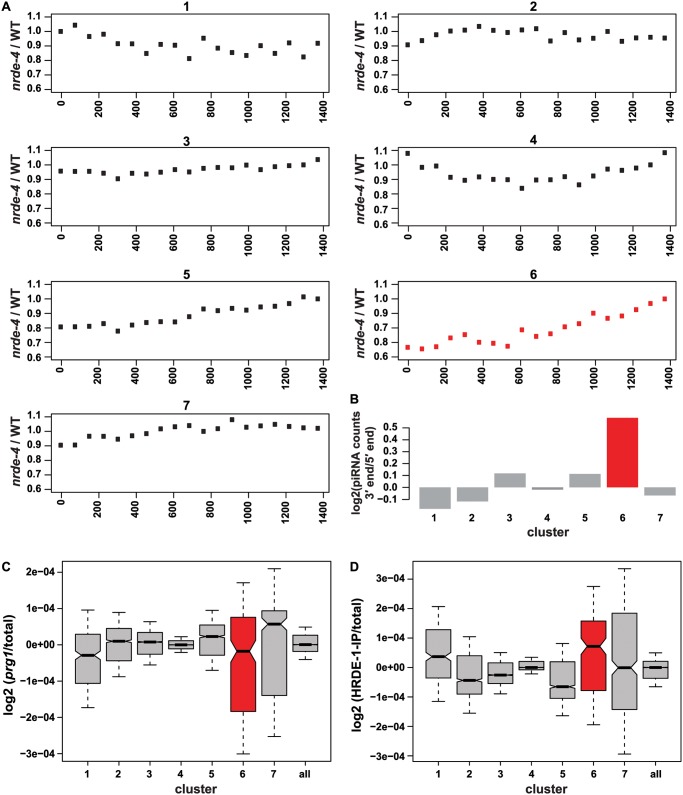
Endogenous 22G-RNA targets show evidence of *nrde*-dependent 3′-5′ spreading of 22G-RNAs. A) Genes were divided into 7 clusters based on the pattern of 22G-RNAs along the gene in *nrde-4* mutants (see methods). The positions of 22G-RNAs relative to the normalized gene length are on the x-axes (see methods). *y*-axes represent the average abundance of 22G-RNAs relative to transcript position between *nrde-4* and wild type (WT) for each cluster. Cluster 6 showing clear reduction at the 5′ end of the gene in *nrde-4* relative to wild type is shown in red. B) Log2 enrichment of predicted piRNA target sites at the 3′ half relative to the 5′ half of endogenous 22G-RNA target transcripts found in clusters 1–7. Cluster 6 is shown in red. C and D) 22G-RNA levels for each gene in total RNA from *prg-1* mutant animals (C) and anti-HRDE-1 IPs (D) relative to 22G-RNA levels in total RNA from wild type animals. Each box corresponds to one of the clusters 1–7. Boxes show the interquartile range, whiskers show the most extreme point that is no less than 1.5 times the interquartile range and outliers are shown as circles. Cluster 6 is shown in red.

These observations were made using a transgene bearing a single piRNA target site in the 3′UTR. We therefore wanted to test whether proximal and distal 22G-RNAs could be detected at endogenous piRNA targets. We observed a similar pattern of 3′ to 5′ spreading of 22G-RNAs at the endogenous transcript Y48G1BM.5 (Fig. 2). The presence of the distal 22G-RNAs depends on *hrde-1*, *nrde-1* and *nrde-4*, while proximal 22G-RNA reads are also greatly reduced in *prg-1* and *mut-16* (Fig. 2). In contrast, *nrde* mutants do not show a reduction in 22G-RNAs at previously characterized endogenous piRNA targets. Since endogenous piRNA target sites could be distributed throughout the length of each target gene, we hypothesized that detection of proximal and distal 22G-RNAs would only be possible for specific piRNA target genes where there was a higher density of piRNA target sites at one or other end of the gene. Only these targets would show a clear reduction in 22G-RNAs in *nrde* mutants. To test this hypothesis we compared the distribution of 22G-RNAs along the length of all genomic transcripts by normalizing to transcript length. We then used k-means clustering to group all 22G-targeted transcripts into 7 clusters based on the distribution of 22Gs along the gene body in nuclear RNAi defective (*nrde*) and *hrde-1* mutants (see methods) and compared the average abundance of 22G-RNAs as a function of transcript position between wild type and *nrde-4* mutants for each cluster. Cluster 6, containing 186 genes, showed strongly reduced 22G reads at the 5′ end of the transcripts compared to the 3′ end in *nrde-4* and *hrde-1* mutants (Figs. 3A and S2). Many individual genes from cluster 6 showed a pattern of nuclear RNAi dependent small RNA accumulation similar to that observed for the *piRNA sensor* (such as Y48G1BM.5, Fig. 2). This suggested that genes within this cluster might display distal 22G-RNAs similar to those seen in the piRNA sensor at their 5′ ends and proximal 22G-RNAs at their 3′ ends of the gene. This would imply that the sites directly targeted by piRNAs would be more common at the 3′ end within this cluster of genes. To test this we mapped sites predicted to be targeted by known piRNA sequences, allowing up to 3 mismatches [11]. Out of all 7 clusters, only Cluster 6 showed a statistically significant enrichment of piRNA target sites in the 3′ half of the gene compared to the 5′ half (p = 0.03, Fisher’s Exact Test; Fig. 3B). Furthermore, Cluster 6 showed strongly reduced 22G-RNA reads in *prg-1* mutants relative to wild type, (Fig. 3C; p<1e-16, Wilcox signed rank test) and showed enrichment for HRDE-1 immunoprecipitated 22G-RNAs (Fig. 3D), consistent with recognition of these targets *in vivo* by both PRG-1 and HRDE-1. Interestingly, cluster 1 showed modestly reduced 22G-RNAs at the 3′ end in nuclear RNAi mutants (Fig. 3A), an enrichment of piRNA targets in the 5′ half of the gene (Fig. 3B), and also had reduced 22G-RNAs in *prg-1* mutants (p<1e-12, Wilcox signed rank test, Fig. 3C) and was enriched for HRDE-1-immunoprecipitated 22G-RNAs (Fig. 3D). This suggests the nuclear RNAi pathway may also mediate 22G-RNA spreading in a 5 to 3 direction along piRNA target transcripts. We conclude for at least a subset of endogenous piRNA targets 22G-RNAs distal to piRNA target sites are dependent on the nuclear RNAi pathway. This analysis supports the notion that two distinct classes of 22G-RNAs are made against many endogenous piRNA targets, and may play an important role in their regulation.

### The nuclear RNAi pathway promotes generation of tertiary siRNAs

The data above was strongly in support of a multi-stage model for the generation of 22G-RNAs against piRNA targets genome-wide. In such a model, proximal 22G-RNAs generated by the initial target recognition would in turn associate with HRDE-1 to trigger distal 22G-RNAs synthesis, in a manner dependent on the nuclear RNAi proteins NRDE-1, NRDE-2 and NRDE-4. This would classify 22G-RNAs that map across the entire transcript as “tertiary” siRNAs as opposed to secondary siRNAs generated by the initial target recognition event [18]. However, it is also possible that both classes are directly dependent on the initial target recognition event and occur in parallel. We therefore generated a reporter gene to distinguish between these two models.

**Fig 4:**
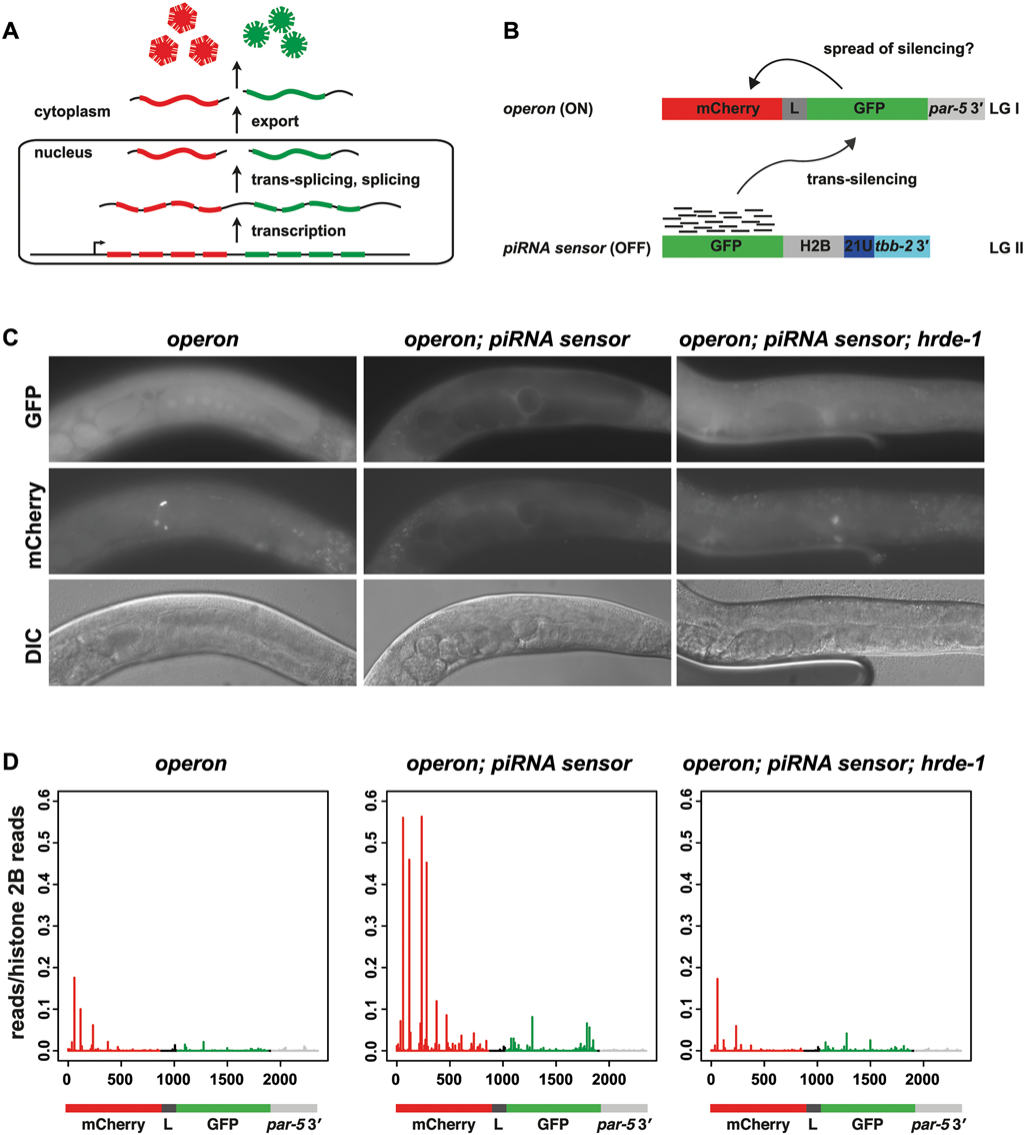
Nuclear RNAi is necessary to initiate synthesis of tertiary siRNAs. A) The principle of the *operon* transgene expression. Straight line represents the DNA locus (bottom), curved lines pre-mRNA and mRNA (before and after nuclear export). Diamonds and snowflakes are mCherry protein (red) and GFP protein (green), respectively (top). B) Schematic representation of the transgenes in the *operon; piRNA sensor* strain. The *piRNA sensor* (bottom) generates 22G-RNAs (black lines) against GFP that can confer trans-silencing of the *operon*-derived GFP. In case a spreading of 22G-RNAs occurs in the nucleus, silencing of mCherry would be expected. C) Representative fluorescence images of somatic and germline GFP (top row) and mCherry (middle row) expression and DIC images (bottom row) of the parental *operon* strain (left), silenced wild type *operon; piRNA sensor* animals (middle) and de-silenced *operon; piRNA sensor; hrde-1* mutant (right) animals. D) Small RNA high-throughput sequencing reads with unique matches antisense to the *operon* from animals as indicated in C). The values of the *y*-axes correspond to reads matching the *operon* normalised to reads matching Histone 2B (*his-58*). The x-axes represent the relative position of reads in the *operon* transgene with numbers representing nucleotides from the start codon (set as 0). The transgene structure is schematically represented at the bottom. Colour code: red = mCherry, dark grey = *gpd-2* trans-splicing linker (L), green = GFP, light grey = *par-5* 3′UTR.

We took advantage of our previous observation that the piRNA-dependent 22G-RNAs present in animals carrying the *piRNA sensor* can trigger trans-silencing of a second GFP-encoding transgene that lacks a perfectly complementary piRNA target site [3]. This effect is mediated by siRNAs antisense to GFP, since a control cross to a similar mCherry-expressing transgene did not result in silencing (S1 Table). We designed a transgenic operon that ubiquitously expresses both mCherry and GFP mRNAs from a single primary transcript, placing mCherry upstream of GFP within the operon (Fig. 4A and 4B; hereafter referred to as the *operon*). Animals carrying a single-copy insertion of the *operon* transgene on chromosome I express both mCherry and GFP throughout development, in both somatic and germline tissues (Fig. 4C). Expression of this transgene is stable and we did not observe any germline silencing in animals carrying the *operon* alone. Since the *piRNA sensor* gives rise to 22G-RNAs antisense to GFP we expected to observe silencing of GFP expression from the *operon* in animals carrying both the *operon* and the *piRNA sensor*. Further, if nuclear RNAi promotes spreading of siRNAs along the length of the target transcript, even in the absence of a perfectly complementary piRNA target site, we would expect expression of mCherry from the *operon* to be silenced, with concomitant synthesis of tertiary 22G-RNAs against to the mCherry coding sequence. Any such silencing and siRNA spreading must be triggered in the nucleus, since the mCherry and GFP mRNAs are exported to the cytoplasm separately (see schematic Fig. 4A and 4B).

Animals homozygous for the *operon* and the *piRNA sensor* (a single-copy insertion on chromosome II), showed germline-specific silencing of GFP expression from the *operon*, confirming that the *piRNA sensor*-derived 22G-RNAs antisense to GFP are able to recognize the *operon*’s GFP-encoding sequence *in trans*. We also observed germline-specific silencing of mCherry expression (Fig. 4C). Trans-silencing of the operon by 22G-RNAs derived from the *piRNA sensor* results in the synthesis of 22G-RNAs antisense to the mCherry coding sequence that are not normally found in animals carrying the *operon* transgene alone (Fig. 4D). Since silencing of the operon transgene is not elicited by direct piRNA targeting, but by secondary 22G-RNAs derived from the *piRNA sensor*, these new 22G-RNAs antisense to mCherry must be tertiary siRNAs. The fact that spreading occurs across distinct transcripts within an operon indicates that tertiary siRNA synthesis is initiated in the nucleus, consistent with a role for the nuclear RNAi machinery. Consistent with the notion that nuclear 22G-RNAs elicit silencing, expression of both GFP and mCherry is restored in *operon*; *piRNA sensor* animals lacking *hrde-1* (Fig. 4C), *mut-16*, *nrde-1*, or *nrde-4* (S3A Fig). Along with the loss of trans-silencing, these animals also fail to generate tertiary 22G-RNAs antisense to the mCherry coding sequence (Figs. 4D and S3B), confirming that nuclear RNAi is necessary to initiate synthesis of this population of tertiary siRNAs. It is important to note that although secondary 22G-RNAs and nuclear RNAi promote tertiary 22G-RNA biogenesis, these data do not indicate the site of (presumably RdRP-dependent) synthesis of new tertiary 22G-RNAs.

These data demonstrate that secondary 22G-RNAs initiated by a piRNA target site can promote the synthesis of tertiary 22G-RNAs, and suggest secondary 22G-RNAs act in the nucleus and independently of the initial piRNA target site to promote tertiary siRNA biogenesis. These data therefore provide an explanation for the nuclear RNAi-dependent spreading of 22G-RNAs across piRNA target loci. The 22G-RNAs that silence piRNA targets, at least at loci with a single piRNA target site, can be divided into secondary and tertiary classes based on the following criteria: secondary 22G-RNAs are found proximal to the piRNA target site, and their biogenesis and stability does not require nuclear RNAi; tertiary siRNAs are found distal to the piRNA target site and are lost in nuclear RNAi-defective mutants. In the case of the *piRNA sensor*, the proximal and distal 22G-RNAs we identified are secondary and tertiary 22G-RNAs respectively. Nuclear RNAi mutants do not generate tertiary siRNAs and fail to silence the *piRNA sensor*, or to trans-silence the GFP-encoding *operon*, suggesting either that both secondary and tertiary siRNAs are required for efficient silencing or that tertiary siRNA generation itself is somehow coupled to the silencing process. Altogether, these data define a new class of tertiary siRNAs and suggests a new mode of small RNA amplification important for repression of piRNA targets.

### Formation of tertiary 22G-RNAs mediates stable transgenerational gene silencing

**Fig 5:**
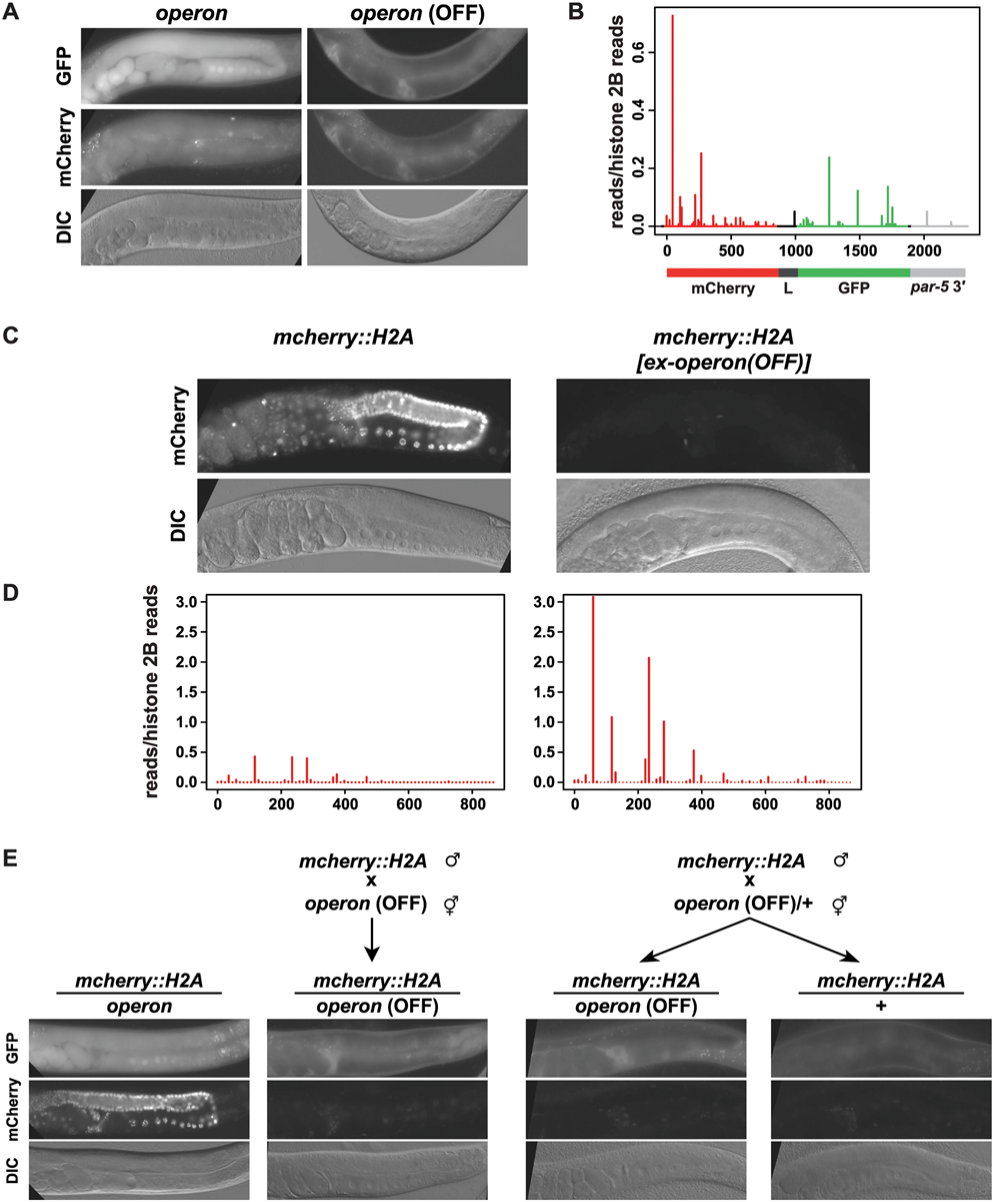
Tertiary 22G-RNAs mediate paramutation. A) Representative fluorescence images of somatic and germline GFP (top row) and mCherry (middle row) expression and DIC images (bottom row) of the parental *operon* strain (left) and the outcrossed, silenced wild type *operon* animals (right). B) Small RNA high-throughput sequencing reads with unique matches antisense to the *operon* from outcrossed *operon* animals. The values of the *y*-axes correspond to reads matching the *operon* normalised to reads matching Histone 2B (*his-58*). The x-axes represent the relative position of reads in the *operon* transgene with numbers representing nucleotides from the start codon (set as 0). The transgene structure is schematically represented at the bottom. Colour code: red = mCherry, dark grey = *gpd-2* trans-splicing linker (L), green = GFP, light grey = *par-5* 3′UTR. C) Fluorescence images of germline mCherry expression (top panels) and DIC images (bottom panels) of the parental *mCherry*::*H2A* (left) and the outcrossed, trans-silenced wild type *mCherry*::*H2A* strains (right). D) Small RNA high-throughput sequencing reads with unique matches antisense to the mCherry transgene from animals as indicated in C). The values of the *y*-axes correspond to reads matching the mCherry sequence normalised to reads matching Histone 2B (*his-58*). The x-axes represent the relative position of reads in the *mCherry*::*H2A* transgene with numbers representing nucleotides from the start codon (set as 0). E) Crossing schemes (top) to generate trans-silenced *mCherry*::*H2A* animals as indicated above the representative fluorescence (GFP top, mCherry middle) and DIC images (bottom). Left panels are from a control cross using the parental non-silenced *operon* strain. Trans-silencing occurs with or without transmission of a silenced *operon* transgene copy.

Both piRNAs and exogenous dsRNA can initiate multigenerational epigenetic silencing in the germline, which depends on nuclear RNAi, and is associated with maintenance of high levels of 22G-RNAs [3,4]. The tertiary 22G-RNAs we identified provide a mechanism for maintenance of high levels of 22G-RNAs over many generations as they could repeatedly re-engage the nuclear RNAi pathway to trigger synthesis of further HRDE-1 bound 22G-RNAs, thus generating a feed-forward amplification loop that can maintain robust levels of 22G-RNAs independent of the initial trigger. In support of this notion, after outcross of *operon*; *piRNA sensor* animals the *operon* remains silenced in the germline regardless of the presence of the *piRNA sensor* (Fig. 5A). This silencing is highly stable; we did not observe any animals revert to germline expression of the *operon* over more than 12 continuous generations in multiple independent silenced lines. We sequenced small RNA populations in such stably silenced lines, and found that silencing is associated with 22G-RNAs antisense to the mCherry coding sequence (Fig. 5B). Supporting the involvement of a nuclear RNAi-dependent 22G-RNA amplification loop, maintenance of this silencing requires the mutator proteins, nuclear RNAi factors, and HRDE-1 (S4 Fig). In addition to triggering heritable *operon* silencing by the *piRNA sensor*, we also initiated heritable silencing by feeding the *operon*-expressing animals with bacteria expressing GFP dsRNA. RNA interference was effectively induced in the parental generation and led not only to complete silencing of the targeted GFP but also to silencing of mCherry. This transgene silencing was maintained over at least 4 generations after progeny of dsRNA-treated animals were removed from RNAi feeding plates. Concomitant with silencing of both the GFP and mCherry, we observed abundant 22G-RNAs antisense to the entire *operon* transcript in the dsRNA-treated P0 and their non-treated F2 generation indicating that exogenous dsRNA can also induce stable heritable gene silencing via a 22G-RNA amplification loop (S5 Fig).

Transitive RNAi or small RNA spreading as a consequence of dsRNA-triggered gene silencing seems to have a strong bias for a 3′ to 5′ direction with regard to the mRNA template [18,21]. However, our analysis of endogenous genes exhibiting nuclear RNAi-dependent spreading of small RNAs would support the notion that spreading could also occur in 5′ to 3′ direction (Fig. 3, cluster 1). To test this directly using the *operon* transgene model, we made use of a second *piRNA sensor* transgene that encodes for a germline-expressed mCherry-Histone 2B fusion protein that also undergoes silencing via the endogenous piRNA 21UR-1 (*mCherry piRNA sensor*, [11]). We repeated the trans-silencing experiment using the *mCherry piRNA sensor* to initiate silencing of the *operon* transgene (S6A Fig). This led to the stable germline-specific silencing of both mCherry and GFP expressed from the *operon*. To investigate whether this silencing was accompanied by 22G-RNA reads mapping to the operon we sequenced small RNAs from silenced strains. We observed abundant small RNAs mapping to both mCherry and GFP portions of the *operon*, indicating that the 5′ to 3′ spreading of small RNAs can occur in this system (S6B Fig).

### Tertiary 22G-RNAs induce paramutation

The stable silencing of the *operon* is reminiscent of paramutation, in which a silent allele initiates epigenetic silencing of an expressed allele [22,23]. The second allele is maintained in a silent state independently of the presence of the initiating allele, and moreover can induce silencing of further expressed alleles, thus itself has become paramutated. This ability to confer paramutation in *trans* is a critical property of paramutated alleles. We tested whether the stably silenced *operon* could act in this manner. We performed crosses between the silenced *operon*, and a number of stably germline-expressed transgenes (Figs. 5C, 5E, S7 and S1 Table). Importantly, we never observed silencing in crosses between any two stably expressed transgenes (Fig. 5E and S1 Table). However, crosses between the silenced *operon* and multiple transgenes bearing the mCherry coding sequence, including the *operon* itself, and a transgene that ubiquitously expresses an mCherry::H2A fusion under the *spn-4* promoter (hereafter *mCherry*::*H2A*) (Fig. 5C and E) resulted in silencing of the expressed allele. After a cross to the silenced *operon*, silencing of the *mCherry*::*H2A* transgene was stably maintained in all descendants over >10 generations, regardless of the presence of the *operon*. This silencing was concomitant with the production of 22G-RNAs that map antisense to the mCherry sequence that are usually not observed in the constitutively expressing parental line (Fig. 5D). Moreover the *mCherry*::*H2A* transgene, like the silenced *operon*, initiated stable silencing of other mCherry encoding transgenes. These observations are consistent with paramutation of the *operon* locus: the *operon* is stably silenced independent of the original trigger (the *piRNA sensor*), and can confer such stable silencing *in trans*.

Surprisingly, despite the robust activity of the silenced *operon* against mCherry expression, it was unable to induce silencing of GFP-expressing transgenes. Indeed, we were able to generate animals carrying both the stably silenced *operon*, and stably germline-expressed GFP::H2B fusions (S7 Fig). This observation is puzzling since silencing of the *operon* was initiated by 22G-RNAs mapping to GFP. However, we note that animals carrying the paramutated *operon* have lower levels of 22G-RNAs antisense to GFP than animals carrying the *piRNA sensor* (Figs. 1A and 5B). The lower levels of 22G-RNAs antisense to GFP derived from the silenced *operon* may be insufficient to initiate silencing. Differences in 22G-RNA levels may result from the sequence differences between the coding regions of the two transgenes leading to different abilities to act as a template for RdRP. It is also possible that other subtle differences in the organization of the transgene, such as promoter strength or choice of 3′ UTR, might lead to differences in their sensitivity to silencing.

### Transmission of silent chromatin is not required for tertiary siRNA-mediated paramutation

Taken together, our observations suggest that a silencing signal associated with the mCherry coding sequence promotes the paramutagenic activity of the stably silenced *operon*. Since we demonstrated that silencing of the *operon* locus requires nuclear RNAi-dependent synthesis of tertiary 22G-RNAs antisense to the mCherry coding sequence, this observation supports the notion that tertiary 22G-RNAs instigate silencing and paramutation. Moreover, the ability of the operon to trigger silencing *in trans* suggests that a diffusible signal is involved. Noting that HRDE-1/22G-RNA complexes are maternally contributed to the zygote, we speculated that inherited HRDE-1/22G-RNA complexes might be sufficient to initiate silencing (and associated 22G-RNA amplification) in progeny independent of the silenced *operon* locus itself. To test this possibility, we crossed hermaphrodites heterozygous for the silenced *operon* with males carrying a stably expressed *mCherry*::*H2A* transgene. We were able to distinguish cross progeny carrying or lacking the silenced *operon* based on somatic expression. We observed the same repression of *mCherry*::*H2A* expression in the germline of cross progeny regardless of inheritance of the silenced *operon* locus (Fig. 5E). From progeny of animals that did not inherit the silenced *operon* locus we were able to isolate *mCherry*::*H2A* transgenic strains that maintained stable multigenerational silencing in the germline (from F1 progeny shown in Fig. 5E, last panel on right). Thus, transmission of silencing does not require inheritance of the silenced locus, implying that altered chromatin at the silenced gene is not pivotal to transmit silencing across generations. Instead, a maternal copy of the silenced *operon* confers multigenerational silencing independent of its own transmission. Thus, our data suggest that inheritance of maternal HRDE-1/22G-RNA complexes is sufficient to confer multigenerational silencing and paramutation. Taken together, these data support a model in which nuclear RNAi-dependent feed forward amplification of tertiary 22G-RNAs is necessary and sufficient for paramutation in animals.

## Discussion

For a molecular signal to carry epigenetic information either through development or between generations, it must be able to template its own regeneration. Without this ability, the signal will become diluted over time as cells divide and the information carried will be lost. Here we show for the first time that small RNAs in *C*. *elegans* can act as such a signal, due to the ability of small RNAs to act upstream of the generation of further, similar small RNAs creating a self-perpetuating loop. Indeed, the ability of this process to generate robust epigenetic states is illustrated by our observation of paramutation such that permanent silencing independent of the initial trigger is established that can in turn instigate further silencing events in a potentially unlimited cycle. Our results provide important insight into how small RNAs can directly promote transgenerational epigenetic inheritance in animals. Here we discuss the implications of our results for the mechanism of 22G-RNA synthesis and the functional consequences of epigenetic inheritance mediated by tertiary siRNAs.

### The mechanism of generation of tertiary siRNAs

Gene silencing by small RNAs in C. elegans is characterised as a two-stage process, by which the primary siRNA:Ago complex recruits RdRPs to a target mRNA that in turn is used as a template for the production of unprimed 22G-RNAs [24,25]. However, so far there has been no evidence for a multi-stage mechanism to generate 22G-RNAs spreading across a target gene.

**Fig 6:**
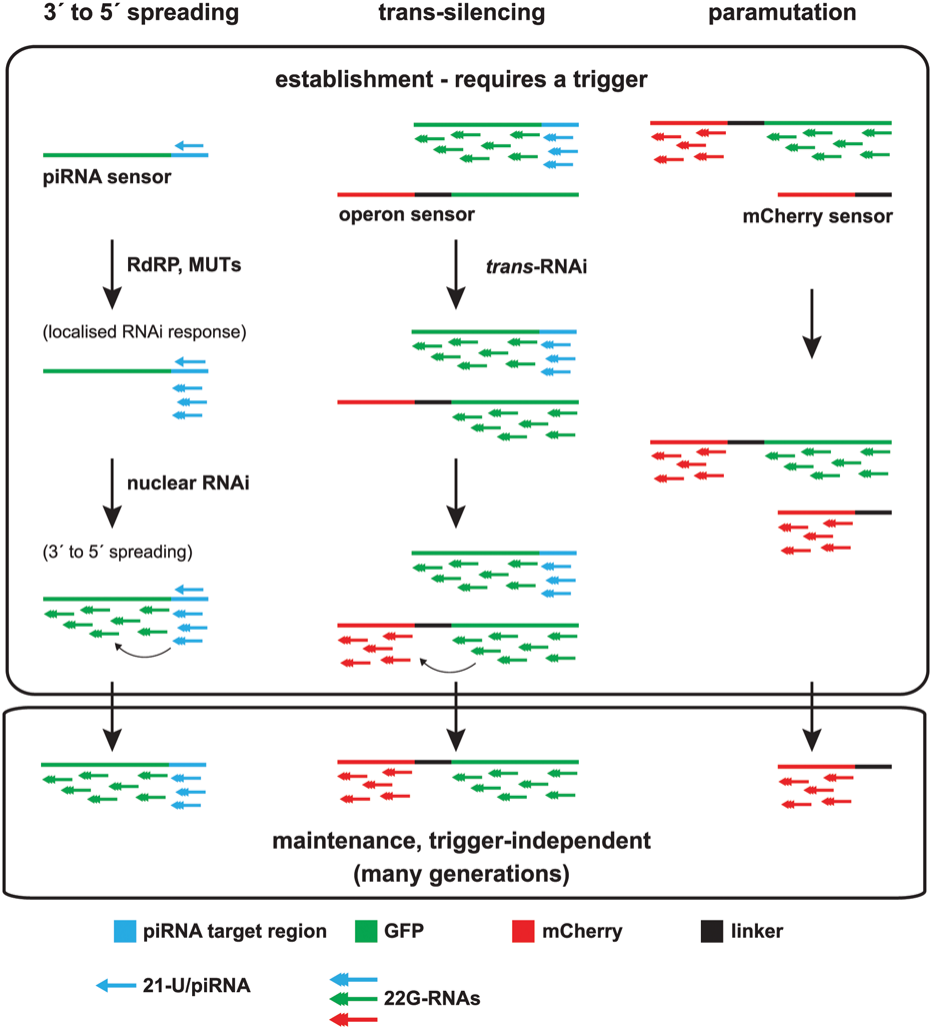
Model of multigenerational target gene silencing by piRNAs and downstream 22G-RNAs. Left: piRNAs against target sites (blue) initiate localized 22G-RNA (blue) production that involves RNA-dependent RNA Polymerases (RdRPs) and Mutator proteins (Muts). 3′ to 5′ spreading of 22G-RNAs along the target gene (e.g. GFP, green) requires the nuclear RNAi pathway. This induces gene silencing that can be maintained over subsequent generations. Middle: Tertiary 22G-RNAs against a target (e.g. GFP, green) are able to silence genes with sequence similarity *in trans*. This leads to further generation of tertiary 22G-RNAs along the trans-silenced target (e.g. the *operon*/mCherry, red) by nuclear RNAi factors. Silencing by tertiary 22G-RNAs can become trigger-independent. Right: Paramutation by tertiary 22G-RNAs against mCherry (red) can be stably maintained in the absence of the original trigger(s).

Here we observed that a subset of 22G-RNAs, which map to regions distal to piRNA target sites on both transgenes and certain endogenous genes, specifically require the nuclear RNAi pathway including the nuclear Argonaute HRDE-1. As these 22G-RNAs are produced only downstream of 22G-RNAs that map closer to piRNA target sites, these can be described as tertiary 22G-siRNAs. Although we cannot formally exclude the possibility that the nuclear RNAi pathway is required only for the stability of tertiary 22G-RNAs, the fact that HRDE-1 binds equally to both classes whilst only being required for the presence of tertiary 22G-RNAs (Fig. 1) suggests that the nuclear RNAi pathway is required for the generation of tertiary 22G-RNAs. In the first stage of this process, secondary 22G-RNAs generated as a direct result of piRNA target recognition bind to the Argonaute HRDE-1 and are transported to the nucleus. Target recognition, in conjunction with the activities of the NRDE proteins NRDE-1, NRDE-2 and NRDE-4, subsequently results in the generation of further, tertiary, 22G-RNAs (Fig. 6). In contrast to the initial 22G-RNA population, tertiary 22G-RNAs are not restricted to the direct vicinity of the piRNA target site, but instead can spread throughout the transcript.

Further experiments will be required to clarify the mode of tertiary 22G-RNA biogenesis. In particular, it is at present unclear how the nuclear RNAi pathway recruits RNA dependent RNA polymerase to the target transcript. One simple possibility is that HRDE-1 directly recruits the RdRP to engage the target transcript. However, we have no evidence for a direct interaction between HRDE-1 and any RdRPs, and the RdRPs previously implicated in 22G-RNA biogenesis localise to the cytoplasm [12]. Moreover, we detected almost no 22G-RNAs against the introns of the transgenes we used in our study (S8 Fig) suggesting that RdRPs act on spliced transcripts, in favour of a cytoplasmic location for tertiary 22G-RNA synthesis. This would require some form of indirect communication between nuclear RNAi factors such as HRDE-1 and the cytoplasmic 22G-RNA biogenesis machinery. The exact mechanism of this communication will be an exciting question for further research.

The ability of tertiary 22G-RNAs to engage the Argonaute HRDE-1 and to instigate the generation of further 22G-RNAs was implied by the detection of abundant 22G-RNAs against germline-expressed transgenes silenced by piRNA targeting that remained in the absence of the initial piRNA itself [3,4]. However, seemingly in contradiction to these observations, in RNAi feeding experiments using dsRNA matching to ubiquitously expressed endogenous genes, careful analysis of deep sequencing data did not give evidence for abundant tertiary siRNAs [18]. Our data enables us to offer a resolution to this paradox.

Here we show that the activity of nuclear RNAi in the germline enables secondary 22G-RNAs, produced as a direct result of piRNA target recognition, to generate tertiary 22G-RNAs. Thus we suggest that the very low abundance of tertiary siRNAs in the RNAi feeding experiments described by Pak *et al*. (2012) may reflect a lack of significant germline nuclear RNAi activity. This would be consistent with the fact that germline nuclear RNAi is not generally required for the efficacy of RNAi by feeding whilst the trigger is present [13], and with the fact that RNAi targeting genes expressed in the soma is only rarely inherited for more than one generation [26]. It seems that, in addition to piRNA targeting, a subset of exogenous RNAi triggers are able to engage the germline nuclear RNAi pathway. Further work will be required to decipher the rules that govern which triggers end up in the nuclear RNAi machinery. This may relate to how some endogenous genes may be protected from targeting by the piRNA pathway as discussed further in the next section.

### Functional implications of tertiary siRNAs

As tertiary siRNAs can instigate the formation of further 22G-RNAs they could lead to potentially unlimited loops of silencing. Indeed we observed such effects in demonstrating paramutation whereby a silenced transgene can convert an expressed transgene into a silent one that in turn has the ability to silence further transgenes. A serious risk is that such an amplification system could silence genes erroneously through the amplification of off-target effects [18]. One way to reduce the likelihood of this process occurring would be the protection mechanism proposed to prevent most germline genes from becoming targets of the piRNA pathway [27,28]. The protection proposed involves the Argonaute CSR-1, which binds to 22G-RNAs antisense to nearly all germline expressed genes, but rather than triggering silencing, prevents targets from being shut down, possibly by antagonizing HRDE-1. In the absence of CSR-1 activity, HRDE-1 activity dominates leading to loss of expression [27,28]. Thus the formation of tertiary 22G-RNAs would only be able to occur at genes that are not targets of CSR-1, such as foreign transgenes and certain endogenous genes including those we identify as being subject to NRDE-dependent siRNA spreading along the transcript. Similarly, RNAi-induced silencing of endogenous CSR-1 targets would be limited to short-term effects and would not engage HRDE-1 and thus would not result in tertiary 22G-RNA synthesis.

Another layer of protection against unchecked silencing once it is established could be through restrictions in successive 22G-RNA production cycles. Given the many endogenous loci targeted by HRDE-1-associated 22G-RNAs, this could likely occur due to competition for a limited pool of factors required for 22G-RNA biogenesis and/or stability such as the RdRPs or Argonaute proteins eventually preventing their uncontrolled amplification.

Given the fact that many *C*. *elegans* genes are protected from stable silencing, it is possible that the multigenerational silencing mediated by HRDE-1/22G-RNAs may only apply to transgenes such as the ones that we use in this study. However, here we describe a set of endogenous genes that behave similarly to the *piRNA sensor* such that 22G-RNAs mapping across the transcript are reduced specifically in mutants lacking the nuclear RNAi pathway whilst those in the vicinity of predicted target sites are maintained. Notably, the genes that fit into this category may represent only a fraction of the genes that are dependent on HRDE-1 for silencing, because 22G-RNAs mapping to HRDE-1 targets would not be expected to be affected if the piRNA target sites map along the entire gene rather than in one place. Recently, Ni *et al*. reported the identification of a set of HRDE-1 target genes that were subject to transcriptional silencing including the accumulation of repressive histone modifications [29]. In accordance with our prediction that 22G-RNAs may not be affected despite dependence on HRDE-1 for silencing, a subset of the genes identified by Ni *et al*., showed little reduction in 22G-RNAs in *hrde-1* mutants [29]. Thus we suggest that some endogenous genes may be subject to transgenerational silencing in addition to transgenes.

Although changes in gene expression that could result from HRDE-1-dependent silencing of endogenous genes may be limited to the germline, this could be linked by systemic signalling to effects in the soma [30,31]. As such, it could provide a mechanism for the transgenerational inheritance of developmental or physiological traits in response to environmental triggers, for example starvation, elevated temperature, or exposure to pathogens. In the harsh world of rotting apples inhabited by *C*. *elegans*, many environmental changes occur on a slower timescale than *C*. *elegans*’ fast reproductive cycle, so ‘Lamarkian’ epigenetic inheritance could be advantageous.

## Materials and Methods

### Nematode culture and strains

We grew *C*. *elegans* under standard conditions at 20°C unless stated otherwise. The wild type strain was var. Bristol N2 [32]. The food source used was *E*. *coli* strain HB101 (*Caenorhabditis* Genetics Center, University of Minnesota, Twin Cities, MN, USA). We used bleaching followed by starvation-induced L1 arrest to generate synchronized cultures. Detailed information about genetic crosses and a list of all strains generated and used in this study are in the Supporting Information (S1 Text).

### DNA constructs and transgenics

Detailed information is provided in the Supporting Information (S1 Text).

### RNAi by feeding

RNAi experiments for transgenerational inheritance were essentially performed as described [3]. Briefly, three L4 larvae were plated onto RNAi plates seeded with either empty vector (pL4440) or GFP dsRNA expressing HT115(DE3) bacteria and allowed to produce progeny. Efficiency of RNAi in this progeny was monitored by GFP fluorescence microscopy. Adult animals were removed after 5 days of initiating RNAi and transferred to non-RNAi plates. Progeny of these animals were sequentially transferred for 3 more generations and monitored for GFP expression. For high-throughput small RNA sequencing, ca. 50 animals from the P0 (treated) or the F2 (non-treated) generations were picked and subjected to RNA extraction.

### Microscopy

We carried out differential interference contrast (DIC) and fluorescence imaging by standard methods [33] using a ZEISS AX10/Imager. A1 upright microscope with 20x objective magnification. Images were taken using an ORCA-ER Digital Camera (Hamamatsu) and processed using Openlab 5 image software (Improvision) and Fiji/ImageJ (version 1.48d).

### Protein extracts preparation, immunopreciptitation and western blotting

Synchronised animals were grown in liquid culture under standard conditions using HB101 as food source at 20°C and collected as young adults to gravid animals. Bacteria were washed off by several washes with M9. Animals were washed and transferred into lysis buffer (20 mM Tris/Cl, ph 8.0, 140 mM KCl, 1.8 mM MgCl2, 0.5% NP-40, 1 mM DTT, 0.1 mM PMSF, Protease Inhibitor Cocktail). A 1:1 slurry of animals in lysis buffer was snap frozen in liquid nitrogen, thawed and homogenized using 0.7mm diameter Zirconia beads (BioSpec products) in a Precellys 24 homogenizer (Bertin Technologies) at 6500 rpm for 2x 20 seconds.

Cell debris was removed by centrifugation and 20 mg of cleared extracts was subjected to HRDE-1 immunoprecipitation using 2.5 μg anti-HRDE-1 polyclonal antibodies [3]. Antibody-antigen complexes were recovered using Dynabeads Protein A (Life Technologies). 5% of immune-precipitated material was subjected to SDS-PAGE followed by Western Blotting with anti-HRDE-1 and anti-PRG-1 antibodies [34]. The remaining antibody-antigen complexes were eluted from the Dynabeads by adding 1ml TRIsure reagent (Bioline) to extract RNA. After snap-freezing in liquid Nitrogen, samples were processed according to the manufacturer’s protocol.

### RNA extraction

For total RNA isolation we harvested synchronised young adult and gravid animals from plates by washing with M9. Alternatively, for animals bearing mutations giving rise to sterile homozygous adults, we picked ca. 50 balancer GFP-negative homozygous mutant L4 to young adult animals from a balanced heterozygous population. The same number of age-matched control animals was used alongside for RNA preparations and small RNA library preparations. We pelleted and dissolved animals in 10 volumes of TRIsure reagent (Bioline). After snap-freezing we homogenized animals by five freeze-thaw cycles in liquid nitrogen. We extracted total RNA according to the manufacturer’s protocol.

### Preparation of 5′-independent small RNA libraries and high-throughput sequencing

cDNA libraries were prepared by treating 1–5 μg total RNA or RNA extracted after HRDE-1 immunoprecipitation with 20 Units RNA 5′ polyphosphatase (Epicentre) in a total volume of 20 μl. De-phophorylated RNA was purified by phenol-chloroform extraction and ethanol precipitation according to standard protocols. Subsequent library preparations were performed with the TruSeq Small RNA library kit (Illumina) following the manufacturer’s instructions with exception that 15 cycles of PCR amplification were used. We size-selected cDNA libraries using 6% TBE PAGE gels (Life Technologies) and ethidium bromide staining. Desired sizes of cDNA bands were cut from the gel (between 147 and 157 nt), the gel matrix broken by centrifugation through gel breaker tubes (IST Engineering Inc.), and cDNA eluted with 400 μl of 0.3M Na-Chloride. Further purification of cDNA was by centrifugation through Spin-X 0.22μm cellulose acetate filter columns (Costar) followed by ethanol precipitation. Libraries were sequenced on a MiSeq Benchtop Sequencer or a HiSeq 2500 Sequencer (Illumina).

### Computational analysis of small RNA high-throughput sequencing data

Processing of small RNA sequencing data to obtain alignments to transcripts was essentially as described [35,36]. For analysis of the genome-wide distribution of small RNAs along transcripts, antisense 22Gs were counted in 10 bins evenly spaced along each spliced transcript corresponding to protein-coding genes as annotated in Wormbase (WS190). These data in *nrde-4* mutants were used as the input for k-means clustering. The algorithm was repeated using 2–8 clusters; at 8 clusters the algorithm failed to converge, thus 7 clusters were used for the final analysis. The genes identified in each cluster were then selected in *nrde-4* and wild type and the average number of reads in each bin in *nrde-4*, normalized to total library size, was divided by the average number in wild type, normalized to total library size to provide the relative ratio of *nrde-4* to wild type at each bin. The logarithm of this was plotted in Fig. 3. The total number of antisense 22Gs across the entire transcript, normalized for total library size was also obtained for each gene. These values were used to plot Fig. 3C and 3D. piRNA target prediction was done by selecting for piRNAs that aligned with up to 3 mismatches antisense to transcripts using Bowtie for alignment as described previously [11]. All data analysis was performed in the R programming environment.

## Supporting Information

S1 Text Additional Materials and Methods. Detailed information about genetic crosses, strains generated and used in this study, DNA constructs and transgenics.
(PDF)

S1 Fig Two RNA-dependent RNA polymerases and the Dicer-related helicase DRH-3 are required for the biogenesis of proximal and distal 22G-RNAs against the piRNA sensor. A) Small RNA high-throughput sequencing reads with unique matches antisense to the *piRNA sensor* from animals as indicated. The values of the *y*-axes correspond to reads matching the *piRNA sensor* normalised to reads matching Histone 2B (*his-58*). The x-axes represent the relative position of reads in the *piRNA sensor* transgene with numbers representing nucleotides from the start codon (set as 0). The transgene structure is schematically represented at the bottom. Colour code: green = GFP, light grey = *his-58*, dark blue = piRNA (21UR-1) target site +/- 50 bp, light blue = *tbb-2* 3′UTR. B) Small RNA high-throughput sequencing reads with unique matches antisense to the *mCherry piRNA sensor* from wild type and *rrf-3* mutant animals. Antisense 22G-RNA reads are displayed as explained in A. The transgene structure is schematically represented at the bottom. Colour code: red = mCherry, light grey = *his-58*, dark blue = piRNA (21UR-1) target site +/- 50 bp, light blue = *tbb-2* 3′UTR.
(PDF)

S2 Fig Endogenous 22G-RNA targets show evidence of *nrde-4 and hrde-1*-dependent 3′-5′ spreading of 22G-RNAs. Cluster 6 containing 186 endogenous genes showing reduction at the 5′ end of the gene in *nrde-4* and *hrde-1* relative to wild type. The positions of 22G-RNAs relative to the normalized gene length are on the x-axes (see methods). *y*-axes represent the average abundance of 22G-RNAs relative to transcript position between *nrde-4* or *hrde-1* and wild type (WT) for each cluster.
(PDF)

S3 Fig Tertiary siRNA production requires the 22G biogenesis machinery and nuclear RNAi. A) Representative fluorescence images of somatic and germ line GFP (top row) and mCherry expression (middle row) and DIC images (bottom row) of the silenced wild type *operon; piRNA sensor* animals and de-silenced *operon; piRNA sensor;* mutant animals as indicated. B) Small RNA high-throughput sequencing reads with unique matches antisense to the *operon* from animals as indicated in A). The values of the *y*-axes correspond to reads matching the *operon* normalised to reads matching Histone 2B (*his-58*). The x-axes represent the relative position of reads in the *operon* transgene with numbers representing nucleotides from the start codon (set as 0). The small RNA profile of the *operon; piRNA sensor* strain is the same as in Fig. 4D. The transgene structure is schematically represented at the bottom. Colour code: red = mCherry, dark grey = *gpd-2* trans-splicing linker (L), green = GFP, light grey = *par-5* 3′UTR.
(PDF)

S4 Fig Stable trans-generational silencing requires nuclear RNAi factors and 22G-RNA biogenesis. Fluorescence images of somatic and germ line GFP (top row) and mCherry expression (middle row) and DIC images (bottom row) of the parental *operon* strain (left), outcrossed wild type *operon* animals (second from left) and de-silenced *operon; mutant* animals as indicated.
(PDF)

S5 Fig Heritable gene silencing and tertiary siRNA production can be induced by exogenous RNAi. A) Small RNA high-throughput sequencing reads with unique matches antisense to the *operon* from non-silenced animals (empty vector, left), GFP dsRNA-treated animals (P0, middle) and their untreated progeny (F2, right). The values of the *y*-axes correspond to antisense 22G-RNA reads matching the *operon* per million total reads. The scale of the middle panel is different to display all antisense 22G-RNA reads in the parental P0 generation. The x-axes represent the relative position of reads in the *operon* transgene with numbers representing nucleotides from the start codon (set as 0). The transgene structure is schematically represented at the bottom. Colour code: red = mCherry, dark grey = *gpd-2* trans-splicing linker (L), green = GFP, light grey = *par-5* 3UTR. B) Antisense 22G-RNA reads per million total reads against either the mCherry or GFP portion of the *operon* were plotted as log2 values to visualise abundance of reads mapping to mCherry and GFP in both the GFP dsRNA-treated P0 and the untreated F2 generation.
(PDF)

S6 Fig Generation of tertiray 22G-RNAs in 5′ to 3′ direction relative to the target mRNA. A) Schematic representation of *piRNA sensor* induced heritable silencing of the *operon* transgene. Left scheme as in Fig. 4. Right scheme depicts the transient and heritable *operon* silencing by the *mCherry piRNA sensor* (both on chromosome I). B) Small RNA high-throughput sequencing reads with unique matches antisense to the *operon* from animals as indicated. The values of the *y*-axes correspond to antisense 22G-RNA reads matching the *operon* per million total reads. The x-axes represent the relative position of reads in the *operon* transgene with numbers representing nucleotides from the start codon (set as 0). The transgene structure is schematically represented at the bottom. Colour code: red = mCherry, dark grey = *gpd-2* trans-splicing linker (L), green = GFP, light grey = *par-5* 3′UTR.
(PDF)

S7 Fig GFP-specific tertiary 22G-RNAs are not capable of inducing paramutation. Fluorescence images of somatic and germ line GFP (top row) and mCherry (middle row) expression and DIC images (bottom row) of two strains carrying the silenced *operon* transgene and non-silenced nuclear GFP-Histone 2B expressing transgenes (*mex-5*::*gfp*::*his-58*::*tbb-2* or *dpy-30*::*his-58*::*gfp*::*tbb-2*, respectively).
(PDF)

S8 Fig Secondary and tertiary 22G-RNAs arise from exons only. Abundance of 22G-RNA reads matching antisense to the *operon* transgene in introns (same for gfp and mcherry) or exons (specific for gfp or mcherry). The values of the *y*-axes correspond to reads per million found per intron or exon, respectively.
(PDF)

S1 Table Summary of genetic crosses of different transgene-carrying strains. Shown are the paternal and maternal genotypes used for the respective crosses and the genotypes of the resulting F1 cross-progeny. TG1 denotes transgene 1 and TG2 denotes transgene 2. Germline expression status is indicated for transgenes 1 and 2, respectively. OFF = no germline expression visible, DIM/OFF = weak germline expression in some animals, ON = germline expression seen in all animals, N/A = not applicable. Transgenes were as follows: *piRNA sensor* = *mjIs144 [mex-5*::*gfp*::*h2b*::*(21UR-1)*::*tbb-2] II*; *operon* = *mjSi67 [dpy-30*::*mcherry*::*gpd-2/3*::*gfp*::*par-5] I*; *mCherry*::*H2A* = *mjSi39 [spn-4*::*mcherry*::*h2a*::*par-5] I*; *H2B*::*GFP* = *mjSi1 [dpy-30*::*h2b*::*gfp*::*tbb-2] II*; *GFP*::*H2B* = *mjIs134 [mex-5*::*gfp*::*h2b*::*tbb-2] II*.
(PDF)
