# Supplementary material for: Tertiary siRNAs Mediate Paramutation in C. elegans
Source: PLoS Genet. 2015 Mar 26;11(3):e1005078. doi: 10.1371/journal.pgen.1005078 (PMC4374809; doi:10.1371/journal.pgen.1005078)
Supplement: S1 Text — Detailed information about genetic crosses, strains generated and used in this study, DNA constructs and transgenics. (PDF) [file pgen.1005078.s001.pdf]

Supporting Information

Materials and Methods

Genetic Crosses.

All strains generated and used in this study are listed in the table below.

Trans-silencing of the operon (mjSi67) by the piRNA sensor (mjIs144).

piRNA sensor (SX1316) males were crossed to *operon* (SX2483) hermaphrodites. After singling the F2 from generation, presence of the two transgenes was confirmed by PCR genotyping assays. To test trans-silencing in various mutant backgrounds, *piRNA sensor*; *operon* (SX2495) males were crossed to various mutants, where possible using a mutant strain already carrying the *piRNA sensor*. After singling the F2 from generation, the presence of the mutation in question and of the two transgenes was confirmed by PCR genotyping assays. Except in the case of *nrde-1*(*gg088*) and *nrde-4*(*gg129*), where the mutants were identified by failure to silence the *piRNA sensor*.

Maintenance of silencing of the operon after outcross of operon; piRNA sensor animals.

Wild type (N2) males were mated with *operon*; *piRNA sensor* (SX2495) hermaphrodites to generate *operon*/+; *piRNA sensor*/+ males. These males were then crossed to wild type hermaphrodites, and *operon*/+ and cross progeny were picked based on somatic expression of GFP and mCherry under a fluorescence dissecting microscope. PCR genotyping was used to identify F2s that lacked the piRNA sensor transgene (*operon*/+ I; + II animals). F3 animals were singled from two such F2 plates, and F3 animals homozygous for the *operon* selected, to generate the strains SX2535 and SX2536. In both of these strains germline expression of the *operon* is stably silenced.

To test genetic requirements for maintenance of silencing, silenced *operon* males (SX2535) were crossed to various mutants. F1 cross progeny were picked by somatic GFP and mCherry expression. F1s were allowed to self and single F2 animals picked and genotype determined by PCR genotyping assay or linked visible marker. Mutant animals were examined within 5 generations for loss of germline silencing of the *operon*.

Silencing and maintenance of the operon by mCherry piRNA sensor animals.

mCherry piRNA sensor (SX2170) males were crossed to *operon* (SX2483) hermaphrodites. After confirming trans-silencing in the trans-heterozygous F1 generation, *operon* F2 animals were isolated and identified by somatic *operon* expression and checked for maintenance of germline silencing of the *operon* by fluorescence microscopy.

Trans-silencing and paramutation by the stably silenced operon transgene.

To test the ability of the silenced *operon* transgene to cause silencing *in trans*, *operon*(OFF) males (SX2535 or SX2536) were crossed with hermaphrodites bearing various germline expressed transgenes: *dpy-30::gfp::h2b::tbb-2* (*mjSi1* II, SX1866); *mex-5::gfp::h2b::tbb-2* (*mjIs134* II, SX1263); *spn-4::mcherry::h2a::par-5* (*mjSi39* I, SX2445). F1 animals were examined for trans-silencing. In the case of the transgenes on LG II encoding GFP::H2B, F2 animals were singled and PCR genotyping used to confirm animals homozygous for both *operon*, and the GFP::H2B transgene in question, to generate the strains SX2569 and SX2570. For the mCherry::H2A transgene on LG I, F2 animals were singled and *spn-4::mcherry::h2a::par-5* homozygous animals identified, to generate SX2571. Germline expression of mCherry::H2A is stably silenced in this strain.

operon(OFF) males (SX2535) were crossed with *unc-73(e936) dpy-5(e61)* I (CB2067) hermaphrodites to generate *operon*(OFF)/*unc-73(e936) dpy-5(e61)* hermaphrodites. *spn-4::mcherry::h2a::par-5* males were crossed to these heterozygous hermaphrodite animals. Cross progeny were identified by somatic expression of nuclear mCherry::H2A. *spn-4::mcherry::h2a::par-5/operon* progeny were distinguished from *spn-4::mcherry::h2a::par-5/ unc-73(e936) dpy-5(e61)* by somatic expression of cytoplasmic GFP and mCherry. Animals of each genotype were examined for germline silencing of mCherry::H2A, alongside progeny from a control cross between *spn-4::mcherry::h2a::par-5* males and hermaphrodites bearing the expressed operon transgene. SX2603 and SX2604 were generated by isolating *spn-4::mcherry::h2a::par-5* homozygous progeny from a *spn-4::mcherry::h2a::par-5/ unc-73(e936) dpy-5(e61)* F1 animals. Germline expression of mCherry::H2A is stably silenced in both of these strains.)

**Strains used in this study**		
**strain**	**genotype**	**comment**
CB2067	*unc-73(e936) dpy-5(e61) I*	
EG6699	*ttTi5605 II; unc-119(ed3) III; oxEx1578*	MosSCI [S2]
EG6701	*ttTi4348 I; unc-119(ed3) III; oxEx1580*	MosSCI [S2]
SX1263	*mjIs134 II; unc-119(ed3) III*	*mex-5::gfp::h2b::tbb-2*
SX1316	*mjIs144 II; unc-119(ed3) III*	*piRNA sensor* [11]
SX1405	*ego-1(om84) / hT2 [bli-4(e937) let-?(q782) qIs48] I;III; mjIs144 II*	[11]
SX1432	*rrf-1(ok589) I; mjIs144 II*	[11]
SX1433	*rrf-2(ok210) I; mjIs144 II*	[11]
SX1636	*mut-16(pk710) I; mjIs144 II*	[11]
SX1638	*avr-14(ad1302) rrf-1(neC1) ego-1(om97) / hT2 [bli-4(e937) let-?(q782) qIs48] I;III; mjIs144 II*	[11]
SX1866	*mjSi1 II; unc-119(ed3) III*	*dpy-30::h2b::gfp::tbb-2* [3]
SX1888	*prg-1(n4357) I; mjIs144 II*	[11]
SX1900	*mjIs144 II; nrde-4(mj259) IV*	[3]
SX2000	*mjIs144 II; hrde-1(tm1200) III*	[11]
SX2091	*drh-3(ne4253) I; mjIs144 II*	[11]
SX2170	*mjSi22 I*	*mCherry piRNA sensor* [11]
SX2171	*mjIs144 II; nrde-1(gg088) III*	[3]
SX2445	*mjSi39 I; unc-119(ed3) III*	*mcherry::H2A* (*spn-4::wormcherry::h2a::par-5*)
SX2483	*mjSi67 I; unc-119(ed3) III*	*operon*
SX2495	*mjSi67 I; mjIs144 II; unc-119(ed3) III*	*operon; piRNA sensor*
SX2535	*mjSi67 I; unc-119(ed3) III*	*operon (OFF)*
SX2536	*mjSi67 I; unc-119(ed3) III*	*operon (OFF)*
SX2567	*mjSi67 I; mjIs144 II; hrde-1(tm1200) III*	
SX2569	*mjSi67 I; mjSi1 II; unc-119(ed3) III*	*operon (OFF); dpy-30::h2b::gfp::tbb-2*
SX2570	*mjSi67 I; mjIs134 II; unc-119(ed3) III*	*operon (OFF); mex-5::gfp::h2b::tbb-2*
SX2571	*mjSi39 I; unc-119(ed3) III*	*mcherry::H2A (OFF)*
SX2575	*mjSi67 I; hrde-1(tm1200) III*	
SX2578	*mjSi67 mut-16(pk710) I*	
SX2596	*mjSi67 mut-16(pk710) I; mjIs144 II*	
SX2602	*mjSi67 I; mjIs144 II; nrde-1(gg088) III*	
SX2603	*mjSi39 I; unc-119(ed3) III*	*mcherry::H2A (OFF)*, from crosses with het. *operon (OFF)* line
SX2609	*mjSi67 I; mjIs144 II; nrde-4(gg129) IV*	
SX2657	*mjSi67 I; nrde-2(gg091) II*	
SX2873	*mjSi67 I; unc-119(ed3) III*	*operon* (OFF)
SX2948	*mjSi22 I; rrf-3(pk1426) II*	

DNA constructs and transgenics.

Gateway entry clones containing each of the following were generated by standard techniques: *spn-4* promoter, *dpy-30* promoter [3, main text] *mex-5* promoter [11, main text] *par-5* 3'UTR, *tbb-2* 3'UTR, mCherry::*gpd-2/3::*GFP (*gpd-2/3* indicates sequence found between the *gpd-2* and *gpd-3* genes in the operon CEOPX036, this includes both the *gpd-2* 3'UTR and sequences necessary for trans-splicing of SL2 to generate an operon), mCherry fused to histone 2A (*his-16*), and GFP fused to H2B his-58 [11, main text] were generated by standard techniques.

We generated DNA vectors for injections using the Multisite Gateway Three-Fragment vector construction kit (Life Technologies). All constructs were confirmed by sequencing. To generate transgenic animals, we performed Mos1 mediated single copy transgene insertions (MosSCI) at insertion site *ttTi5605* on chromosome II or at insertion site *ttTi4348* on chromosome I as described [S1,S2]. Injection mixes contained 20 ng μl^{-1} of vector, 20 ng μl^{-1} of Mos1 transposase, and 5 ng μl^{-1} of each marker (pharynx and body wall muscle). Details on cloning and plasmid sequences are available upon request.

Supporting References

S1. Frøkjær-Jensen C, Wayne Davis M, Hopkins CE, Newman BJ, Thummel JM, et al. (2008) Single-copy insertion of transgenes in Caenorhabditis elegans. Nat Genet 40: 1375–1383. doi:10.1038/ng.248.

S2. Frøkjær-Jensen C, Davis MW, Ailion M, Jorgensen EM (2012) Improved Mos1-mediated transgenesis in C. elegans. Nat Methods 9: 117–118. doi:10.1038/nmeth.1865.
