# Supplementary figures and images for: Tertiary siRNAs Mediate Paramutation in C. elegans
Source: PLoS Genet. 2015 Mar 26;11(3):e1005078. doi: 10.1371/journal.pgen.1005078 (PMC4374809; doi:10.1371/journal.pgen.1005078)

Figure S1

A

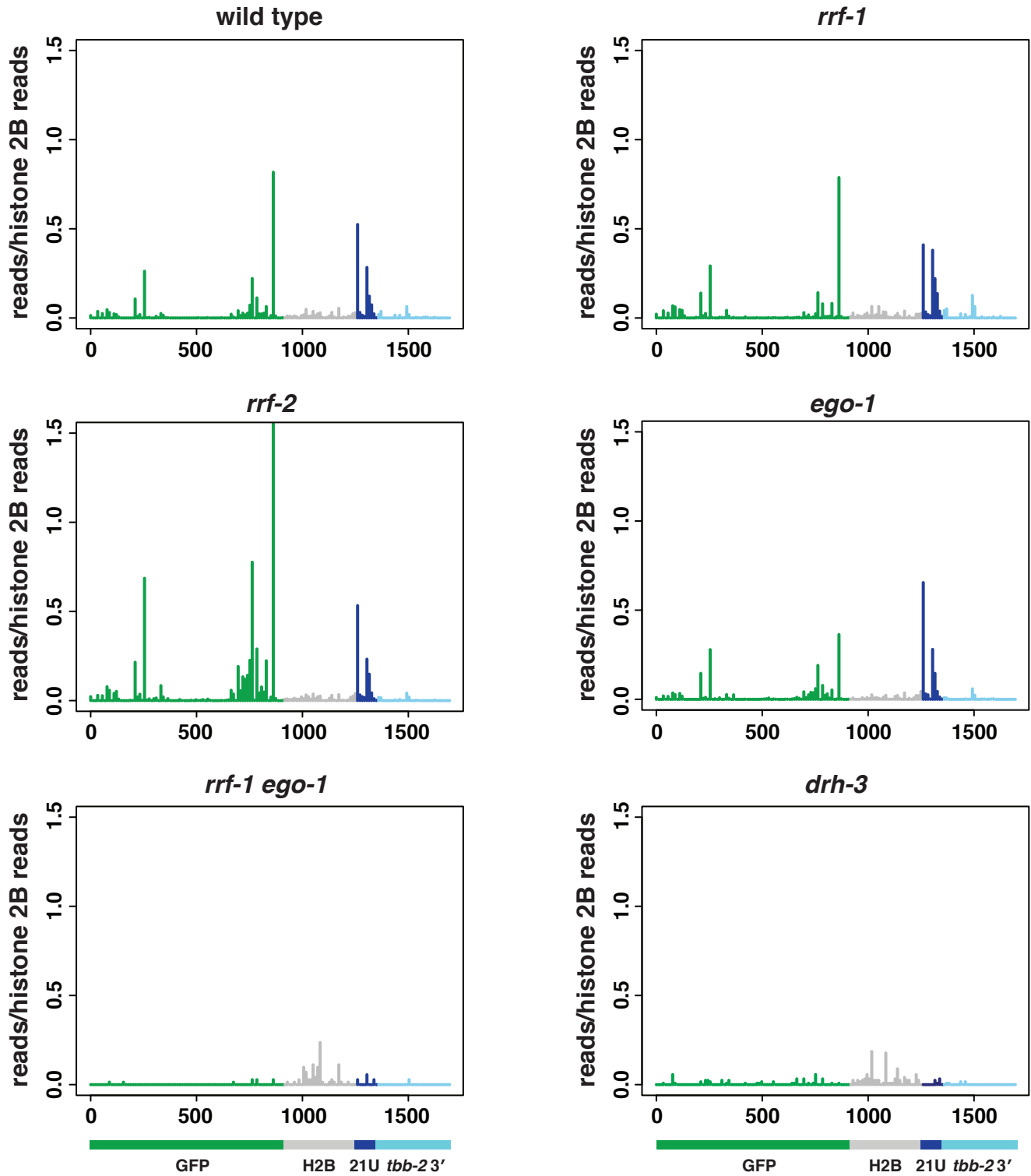

B

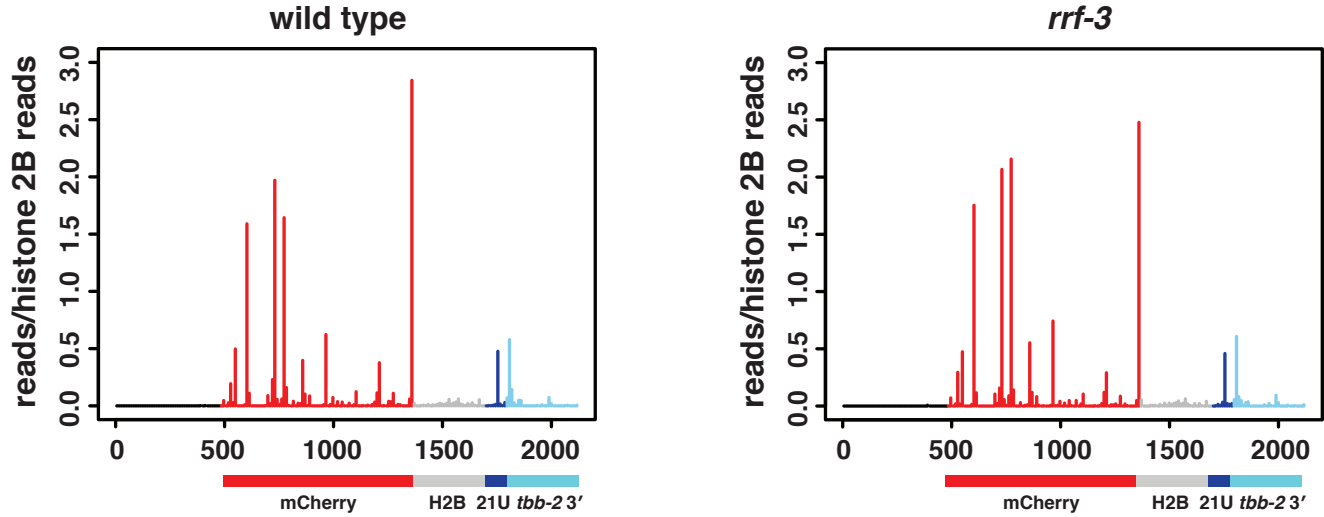

Supplement: S1 Fig — A) Small RNA high-throughput sequencing reads with unique matches antisense to the piRNA sensor from animals as indicated. The values of the y-axes correspond to reads matching the piRNA sensor normalised to reads matching Histone 2B (his-58). The x-axes represent the relative position of reads in the piRNA sensor transgene with numbers representing nucleotides from the start codon (set as 0). The transgene structure is schematically represented at the bottom. Colour code: green = GFP, light grey = his-58, dark blue = piRNA (21UR-1) target site +/- 50 bp, light blue = tbb-2 3′UTR. B) Small RNA high-throughput sequencing reads with unique matches antisense to the mCherry piRNA sensor from wild type and rrf-3 mutant animals. Antisense 22G-RNA reads are displayed as explained in A. The transgene structure is schematically represented at the bottom. Colour code: red = mCherry, light grey = his-58, dark blue = piRNA (21UR-1) target site +/- 50 bp, light blue = tbb-2 3′UTR. (PDF) [file pgen.1005078.s002.pdf]

Figure S2

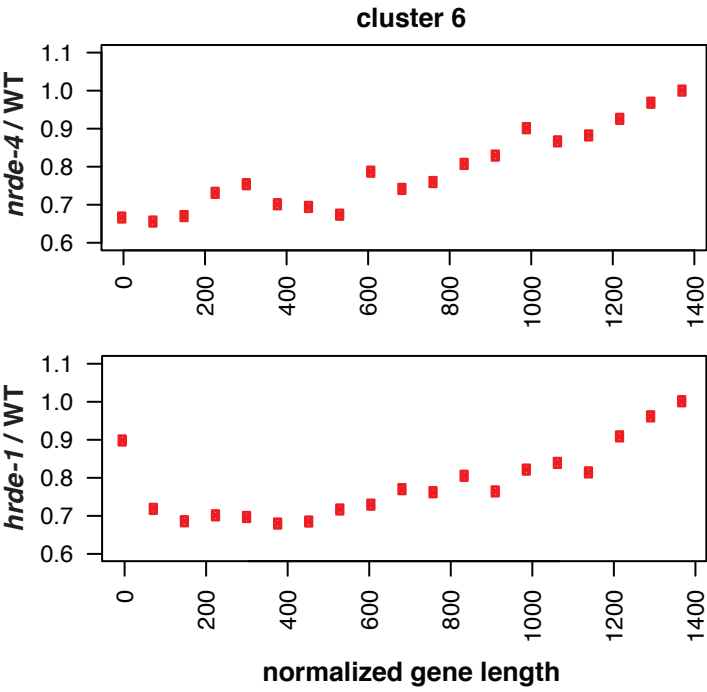

Supplement: S2 Fig — Cluster 6 containing 186 endogenous genes showing reduction at the 5′ end of the gene in nrde-4 and hrde-1 relative to wild type. The positions of 22G-RNAs relative to the normalized gene length are on the x-axes (see methods). y-axes represent the average abundance of 22G-RNAs relative to transcript position between nrde-4 or hrde-1 and wild type (WT) for each cluster. (PDF) [file pgen.1005078.s003.pdf]

**Figure S3**

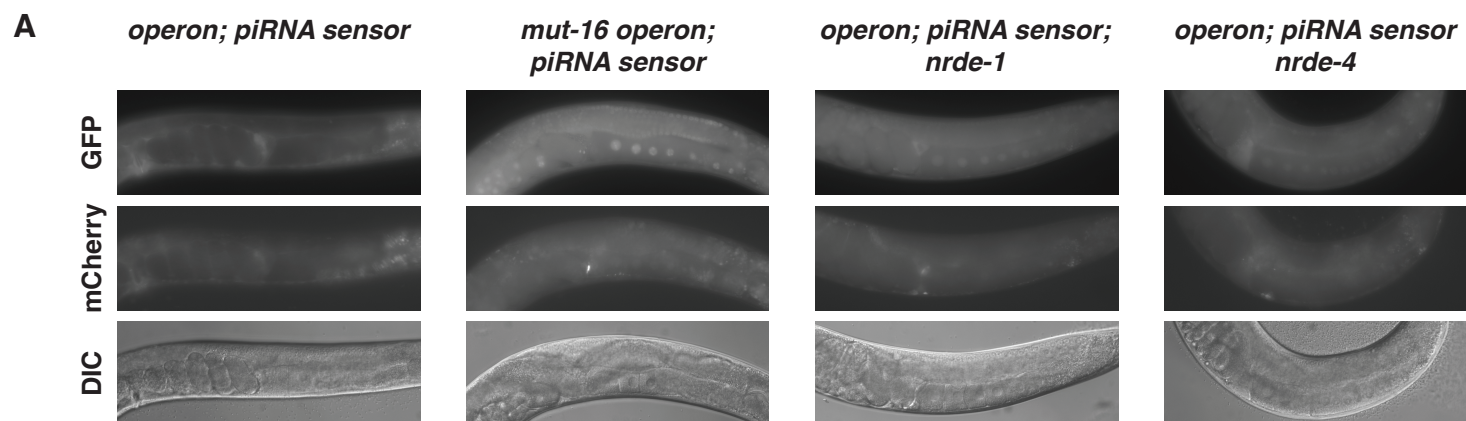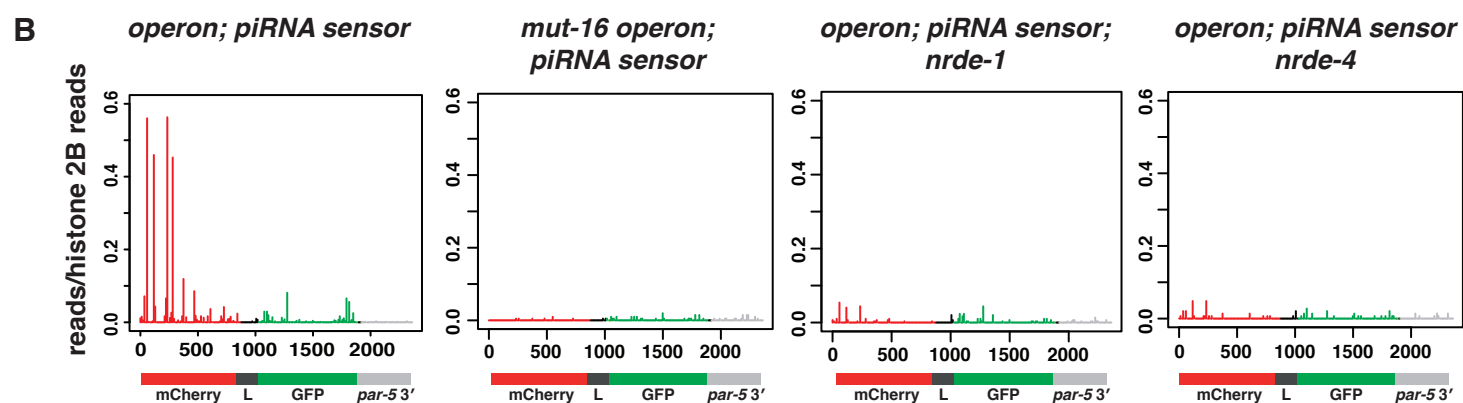

Supplement: S3 Fig — A) Representative fluorescence images of somatic and germ line GFP (top row) and mCherry expression (middle row) and DIC images (bottom row) of the silenced wild type operon; piRNA sensor animals and de-silenced operon; piRNA sensor; mutant animals as indicated. B) Small RNA high-throughput sequencing reads with unique matches antisense to the operon from animals as indicated in A). The values of the y-axes correspond to reads matching the operon normalised to reads matching Histone 2B (his-58). The x-axes represent the relative position of reads in the operon transgene with numbers representing nucleotides from the start codon (set as 0). The small RNA profile of the operon; piRNA sensor strain is the same as in Fig. 4D. The transgene structure is schematically represented at the bottom. Colour code: red = mCherry, dark grey = gpd-2 trans-splicing linker (L), green = GFP, light grey = par-5 3′UTR. (PDF) [file pgen.1005078.s004.pdf]

Figure S4

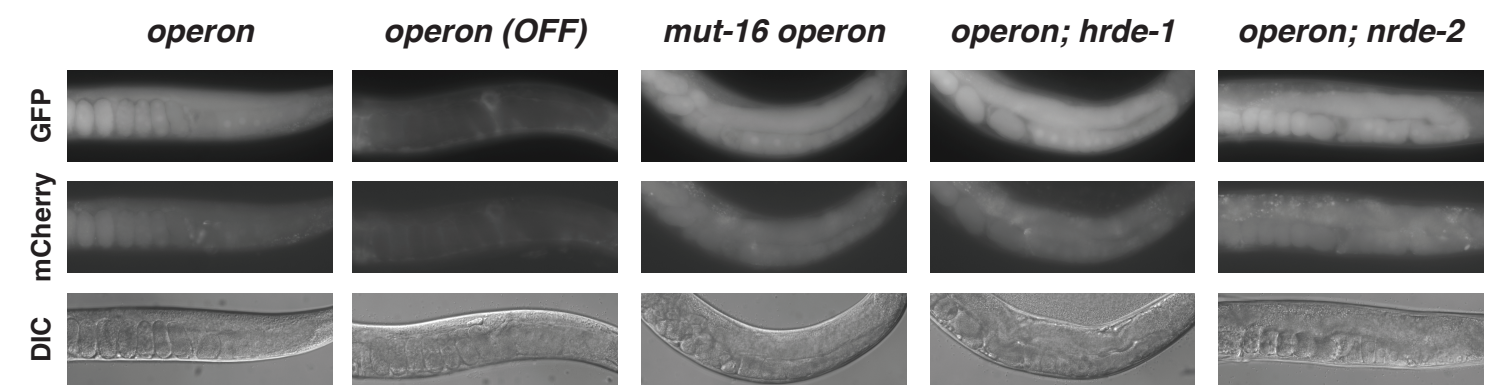

Supplement: S4 Fig — Fluorescence images of somatic and germ line GFP (top row) and mCherry expression (middle row) and DIC images (bottom row) of the parental operon strain (left), outcrossed wild type operon animals (second from left) and de-silenced operon; mutant animals as indicated. (PDF) [file pgen.1005078.s005.pdf]

Figure S5

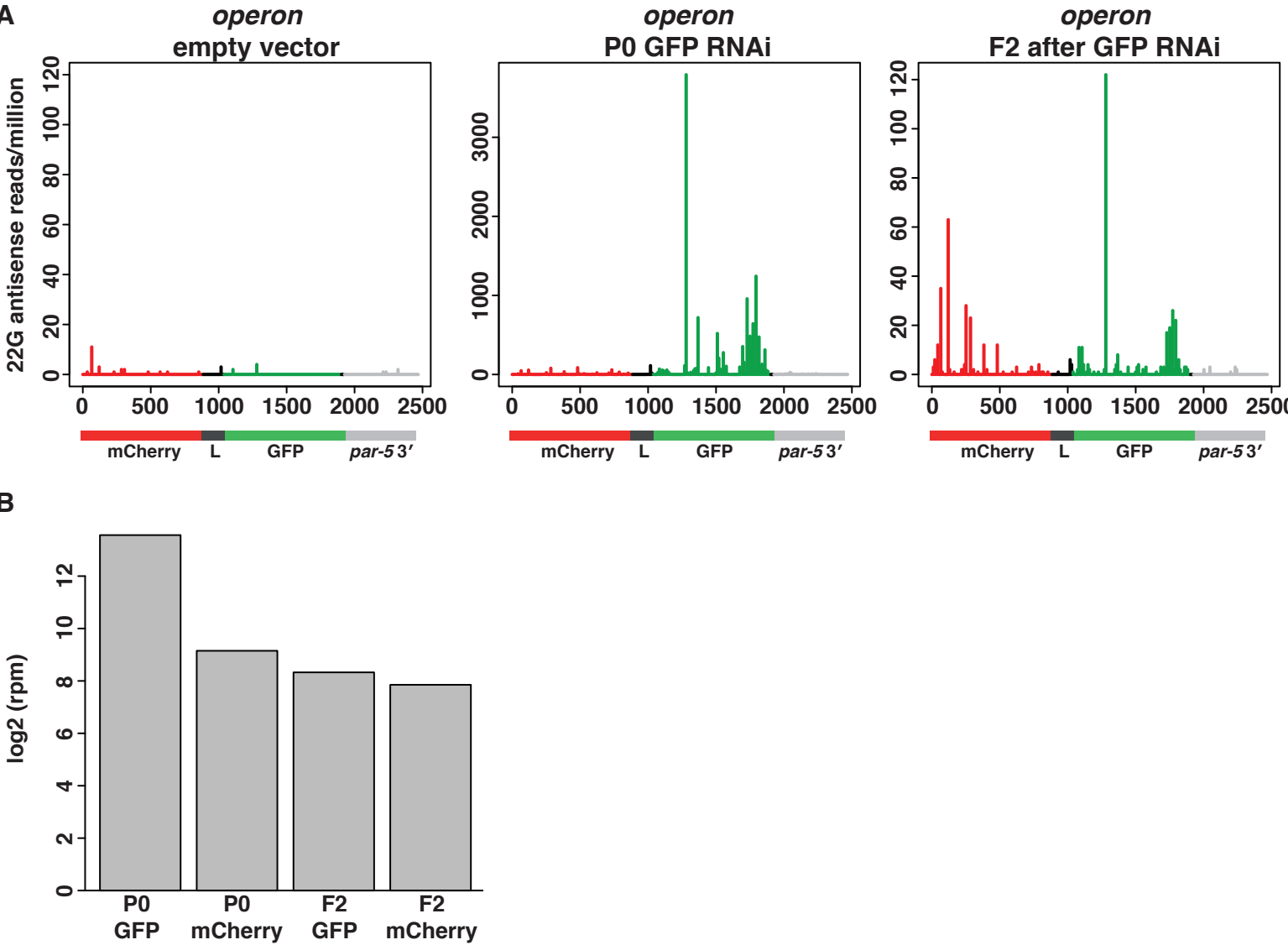

Supplement: S5 Fig — A) Small RNA high-throughput sequencing reads with unique matches antisense to the operon from non-silenced animals (empty vector, left), GFP dsRNA-treated animals (P0, middle) and their untreated progeny (F2, right). The values of the y-axes correspond to antisense 22G-RNA reads matching the operon per million total reads. The scale of the middle panel is different to display all antisense 22G-RNA reads in the parental P0 generation. The x-axes represent the relative position of reads in the operon transgene with numbers representing nucleotides from the start codon (set as 0). The transgene structure is schematically represented at the bottom. Colour code: red = mCherry, dark grey = gpd-2 trans-splicing linker (L), green = GFP, light grey = par-5 3UTR. B) Antisense 22G-RNA reads per million total reads against either the mCherry or GFP portion of the operon were plotted as log2 values to visualise abundance of reads mapping to mCherry and GFP in both the GFP dsRNA-treated P0 and the untreated F2 generation. (PDF) [file pgen.1005078.s006.pdf]

**Figure S6**

**A**

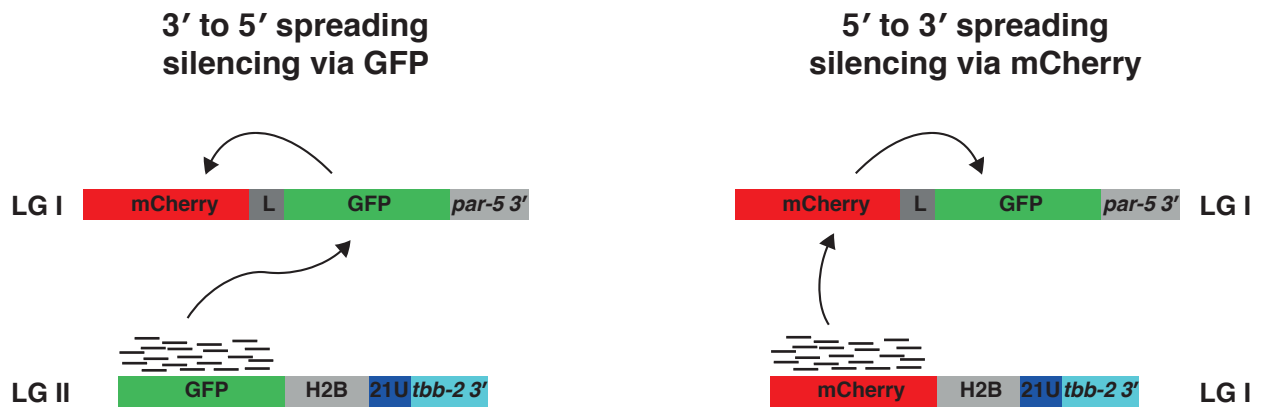

**B**

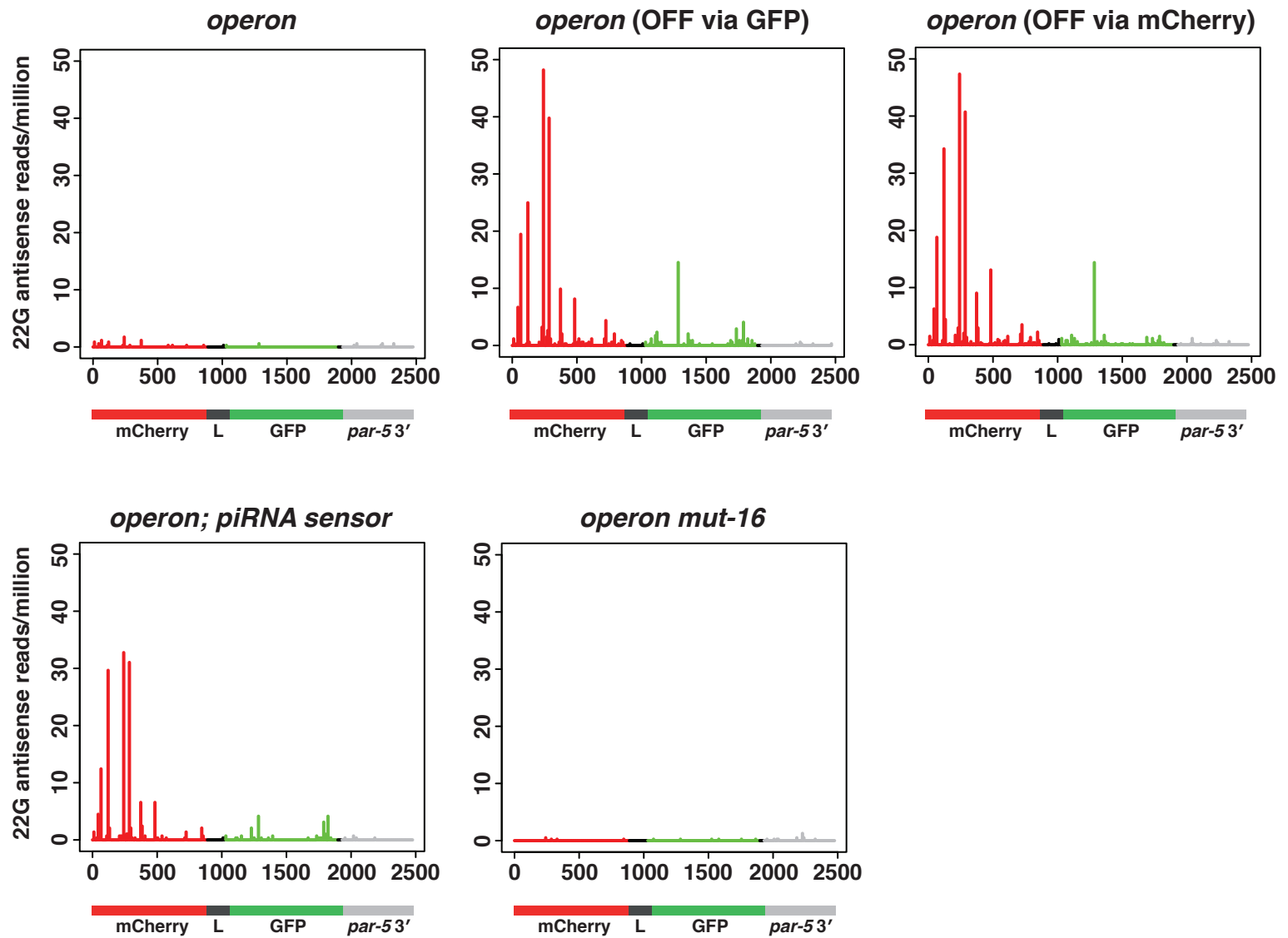

Supplement: S6 Fig — A) Schematic representation of piRNA sensor induced heritable silencing of the operon transgene. Left scheme as in Fig. 4. Right scheme depicts the transient and heritable operon silencing by the mCherry piRNA sensor (both on chromosome I). B) Small RNA high-throughput sequencing reads with unique matches antisense to the operon from animals as indicated. The values of the y-axes correspond to antisense 22G-RNA reads matching the operon per million total reads. The x-axes represent the relative position of reads in the operon transgene with numbers representing nucleotides from the start codon (set as 0). The transgene structure is schematically represented at the bottom. Colour code: red = mCherry, dark grey = gpd-2 trans-splicing linker (L), green = GFP, light grey = par-5 3′UTR. (PDF) [file pgen.1005078.s007.pdf]

**Figure S7**

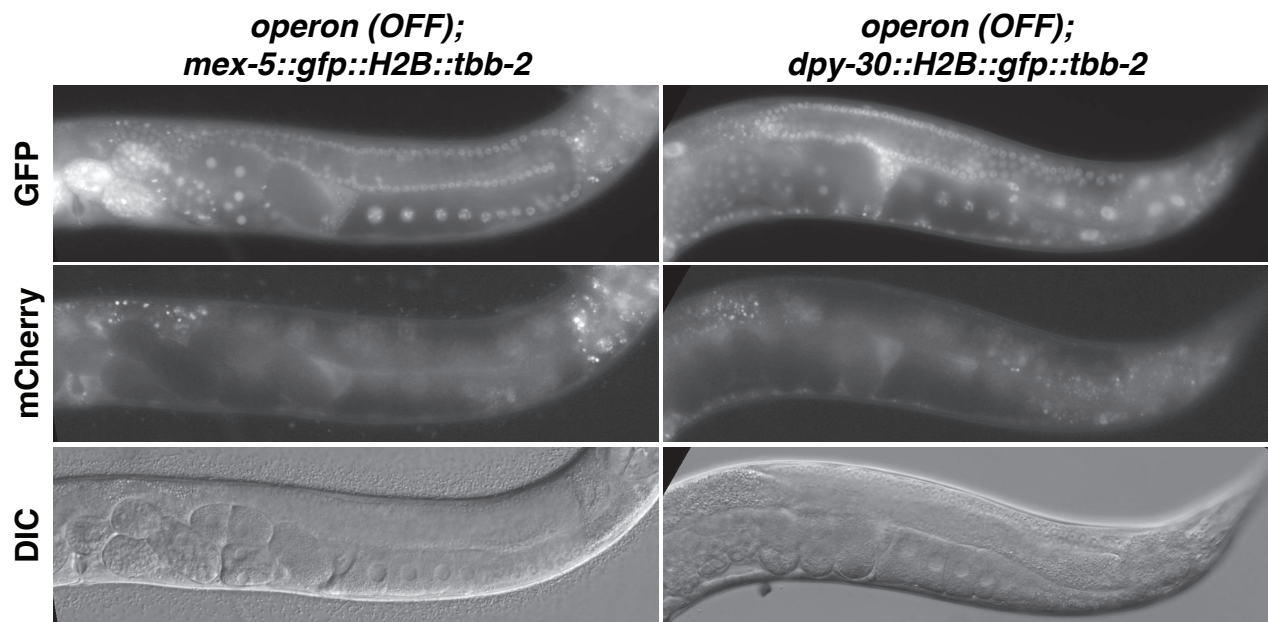

Supplement: S7 Fig — Fluorescence images of somatic and germ line GFP (top row) and mCherry (middle row) expression and DIC images (bottom row) of two strains carrying the silenced operon transgene and non-silenced nuclear GFP-Histone 2B expressing transgenes (mex-5::gfp::his-58::tbb-2 or dpy-30::his-58::gfp::tbb-2, respectively). (PDF) [file pgen.1005078.s008.pdf]

**Figure S8**

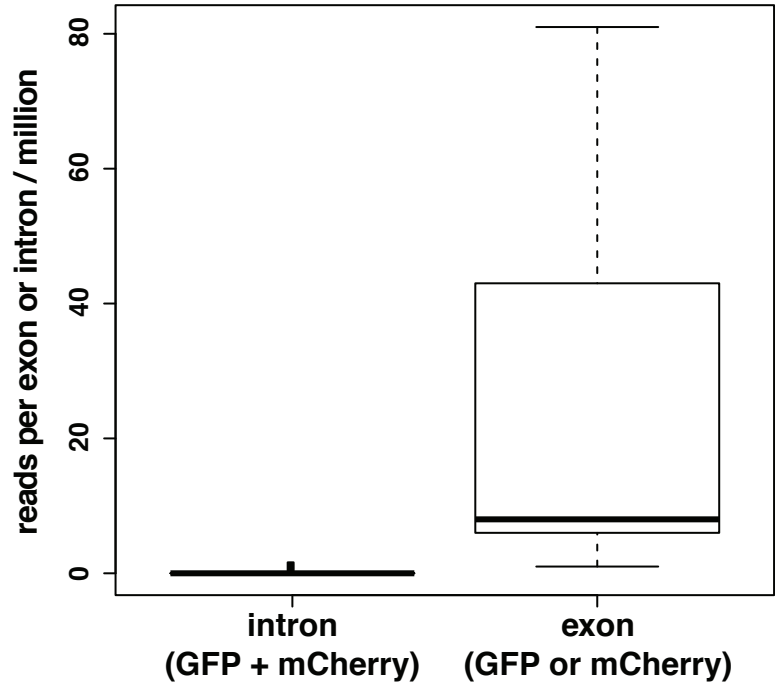

Supplement: S8 Fig — Abundance of 22G-RNA reads matching antisense to the operon transgene in introns (same for gfp and mcherry) or exons (specific for gfp or mcherry). The values of the y-axes correspond to reads per million found per intron or exon, respectively. (PDF) [file pgen.1005078.s009.pdf]
