# Supplementary material for: Tertiary siRNAs Mediate Paramutation in C. elegans
Source: PLoS Genet. 2015 Mar 26;11(3):e1005078. doi: 10.1371/journal.pgen.1005078 (PMC4374809; doi:10.1371/journal.pgen.1005078)
Supplement: S1 Table — Shown are the paternal and maternal genotypes used for the respective crosses and the genotypes of the resulting F1 cross-progeny. TG1 denotes transgene 1 and TG2 denotes transgene 2. Germline expression status is indicated for transgenes 1 and 2, respectively. OFF = no germline expression visible, DIM/OFF = weak germline expression in some animals, ON = germline expression seen in all animals, N/A = not applicable. Transgenes were as follows: piRNA sensor = mjIs144 [mex-5::gfp::h2b::(21UR-1)::tbb-2] II; operon = mjSi67 [dpy-30::mcherry::gpd-2/3::gfp::par-5] I; mCherry::H2A = mjSi39 [spn-4::mcherry::h2a::par-5] I; H2B::GFP = mjSi1 [dpy-30::h2b::gfp::tbb-2] II; GFP::H2B = mjIs134 [mex-5::gfp::h2b::tbb-2] II. (PDF) [file pgen.1005078.s010.pdf]

Table S1, trans-silencing and paramutation testing.

Paternal genotype	Maternal genotype	F1 genotype (TG1/TG2 or TG1/+; TG2/+)	Expression TG1	Expression TG2
piRNA sensor	*operon*	*piRNA sensor/operon*	OFF	OFF
operon	*piRNA sensor*	*piRNA sensor/operon*	OFF	OFF
piRNA sensor	*mCherry::H2A*	*piRNA sensor/+; mCherry::H2A/+*	OFF	ON
mCherry::H2A	piRNA sensor	*piRNA sensor/+; mCherry::H2A/+*	OFF	ON
operon (off)	*operon*	*operon*	DIM/OFF	N/A
operon	*operon* (off)	*operon*	OFF	N/A
operon	*mCherry::H2A*	*operon/mCherry::H2A*	ON	ON
mCherry::H2A	*operon*	*operon/mCherry::H2A*	ON	ON
operon (off)	*mCherry::H2A*	*operon/mCherry::H2A*	OFF	DIM
mCherry::H2A	*operon* (off)	*operon/mCherry::H2A*	OFF	OFF
operon (off)	*H2B::GFP*	*operon/+ ; H2B::GFP/+*	OFF	ON
H2B::GFP	*operon* (off)	*operon/+ ; H2B::GFP/+*	OFF	ON
operon (off)	*GFP::H2B*	*operon/+ ; GFP::H2B/+*	OFF	ON
GFP::H2B	*operon* (off)	*operon/+ ; GFP::H2B/+*	OFF	ON
operon (off)/+	*mCherry::H2A*	*operon/mCherry::H2A*	OFF	DIM
		+/mCherry::H2A	N/A	DIM
mCherry::H2A	*operon* (off)/+	*operon/mCherry::H2A*	OFF	OFF
		+/mCherry::H2A	N/A	OFF
operon (off)	+	*operon/+*	OFF	N/A
+	*operon* (off)	*operon/+*	OFF	N/A
